# Gonadal sex reversal at single-cell resolution in *Znrf3*-deficient mice

**DOI:** 10.1242/dev.202707

**Published:** 2024-12-04

**Authors:** Raissa G. G. Kay, Richard Reeves, Pam Siggers, Simon Greenaway, Ann-Marie Mallon, Sara Wells, Bon-Kyoung Koo, Chloé Mayère, Serge Nef, Andy Greenfield, Michelle M. Simon

**Affiliations:** ^1^ MRC Mammalian Genetics Unit, MRC Harwell Institute, Didcot, Oxfordshire OX11 0RD, UK; ^2^ Mary Lyon Centre at MRC Harwell, Didcot, Oxfordshire OX11 0RD, UK; ^3^ Center for Genome Engineering, Life Science Institute, Institute for Basic Science, 55 Expo-ro, Yuseong-gu, Daejeon, 34126, Republic of Korea; ^4^ Department of Genetic Medicine and Development, Faculty of Medicine, University of Geneva, CH-1206 Geneva 4, Switzerland; ^5^ King's College London, Hub for Applied Bioinformatics, Guy's Hospital, London SE1 9RT, UK

**Keywords:** Genetics of sex, Ovotestis, Sex determination, Sex reversal, Testis, Znrf3, Mouse

## Abstract

The role of anti-WNT ZNRF3 is central to determining gonadal fate: XY mice lacking functional ZNRF3 exhibit a highly variable gonadal sex reversal phenotype in the fetal period, characterised by appearance of ovarian tissue. To investigate this sex reversal further, we used single-cell RNA-seq to examine the transcriptomes of XY *Znrf3*-deficient gonads during the mouse sex-determining period. Analyses of cell trajectories in mutant gonads reveal the failure of pre-supporting cells to commit to the Sertoli cell fate, XY granulosa cell development, unstable commitment in those cells that reach the Sertoli path and enhanced contribution to a supporting-like cell fate. By developing a machine learning-based score for transcriptomic similarity to Sertoli and granulosa, we show pervasive disruption to acquisition of testicular cell fate in the mutant supporting cell lineage, with large numbers of cells co-expressing pro-Sertoli and pro-granulosa markers. These data reveal that loss of *Znrf3* results in transcriptomic and cellular heterogeneity, with shifts in cellular sex identity that undermine a simple binary model in which mutant supporting cell precursors achieve either Sertoli or granulosa cell differentiation.

## Teaser

**Summary:** ZNRF3-deficient embryonic male gonads exhibit pervasive disruption of sex-specific expression leading to unresolved gonadal supporting cell fates.

## INTRODUCTION

Mammalian sex determination is the process by which the bipotential fetal gonad primordium commits to either the testicular or ovarian fate (Greenfield, 2013; Nef et al., 2019). In XY fetuses, testis determination is initiated by the expression of the Y-linked *Sry* gene in supporting cell precursors (Koopman et al., 1991; Hacker et al., 1995). SRY is recruited to enhancers at the *Sox9* locus to promote its upregulation (Gonen et al., 2017, 2018). SOX9 is then recruited to hundreds of genomic targets in order to orchestrate a genetic programme resulting in Sertoli cell differentiation (Rahmoun et al., 2017). This also requires paracrine action of the secreted growth factor FGF9 (Colvin et al., 2001; Hiramatsu et al., 2010). In the absence of Sry, canonical WNT signals direct supporting cell precursors towards a granulosa cell fate (Pannetier et al., 2016). Moreover, in the presence of an overexpressed WT1, transcription factors FOXL2 and RUNX1, were also found to assist supporting cell precursors towards a granulosa cell fate (Nicol et al., 2019; Bridges et al., 2022; Gregoire et al., 2023). The pro-testis and pro-ovary gene regulatory networks are mutually antagonistic: each one acts to oppose the other, canalizing just one developmental pathway, in ways that are slowly becoming clearer (Kim et al., 2006; Greenfield, 2015; Nef et al., 2019).

We have reported a role for the E3 ubiquitin ligase ZNRF3 in testis determination (Harris et al., 2018). ZNRF3 acts to antagonise WNT signalling by increasing membrane turnover of Frizzled receptor (Hao et al., 2012; Koo et al., 2012). By contrast, RSPO1, which is required for normal ovary development in both human and mouse (Parma et al., 2006; Chassot et al., 2008), potentiates WNT signalling by sequestering ZNRF3 and the related protein RNF43, thereby stabilising membrane Frizzled receptor (Zebisch et al., 2013). Nevertheless, questions remain concerning the molecular roles and control of ZNRF3/RNF43 and their interactions with RSPO1 (Giebel et al., 2021; Kim et al., 2021; Tsukiyama et al., 2021). In *Znrf3* mutant homozygotes, XY fetal gonads on the C57BL/6J (B6J) mouse genetic background, a mouse strain sensitised to disruptions to testis determination owing to fewer SRY-expressing cells (Bouma et al., 2007; Narita et al., 2023), exhibit ectopic canonical WNT signalling in the sex-determining period [11.5 days post coitum (dpc)] and associated downregulation of expression of testis-determining *Sox9* at the same stage (Harris et al., 2018). However, this gonadal sex reversal in *Znrf3* mutant fetuses is characterised by profound phenotypic variability: XY mutant gonads can develop as gonads with substantial amounts of testicular tissue, as ovotestes or as morphological ovaries, with variability sometimes seen even within a single fetus.

Here, we describe experiments aimed at analysing sex reversal in *Znrf3* mutants at single-cell resolution, with a focus on the development of the supporting cell lineage, which drives gonadal sex determination. We used single-cell RNA sequencing (scRNA-seq) to examine cellular transcriptomes at three stages of mouse (B6J) gonad development: in the bipotential gonad at the sex-determining stage (11.5 dpc) and in the differentiating fetal gonads, at 12.5 dpc and 14.5 dpc, in three genotypes; XX and XY wild type (WT) and XY *Znrf3* homozygous mutant (XY *Z*-del). We also compared the landscape of sex determination at single-cell resolution in the sensitised B6J strain with the non-sensitised strain CD1 (Mayère et al., 2022), which allowed us to calibrate the nature of sex reversal in the *Z*-del mutants by understanding the ‘feminised’ baseline of testis determination in B6J XY gonads. Analysis of the *Z*-del mutant confirmed both granulosa and Sertoli cell differentiation in individual *Znrf3* mutant gonads, but with altered trajectories of supporting cell precursors, resulting in elevated numbers of precursors that fail to commit to the Sertoli cell fate and maintain a bipotential state, and unstable commitment in those that do. Moreover, we show increased trajectory of some pre-supporting cells and pre-Sertoli cells in *Z*-del mutants towards a recently reported supporting-like cell (SLC) type, characterised by *Pax8* expression. By using a machine-learning algorithm to score the transcriptomic identity of testicular and ovarian supporting cells, we show that there is pervasive disruption to cellular sex identity in *Z*-del mutants, affecting all supporting cell-types. These data indicate that care must be taken when attributing cell identity on the basis of a limited number of markers, especially in the context of mutant gonad development. In addition to revealing a remarkable molecular and cellular heterogeneity in the *Z*-del XY gonadal sex reversal phenotype, often characterised by shifts rather than switches, our data also represent a rich source of information on transcriptional differences between constituent gonadal cell-types and the relationship between these cell lineages throughout the sex-determining period in the sensitised B6J mouse model.

## RESULTS

### Annotation of the gonadal cell clusters

**Fig. 1.**
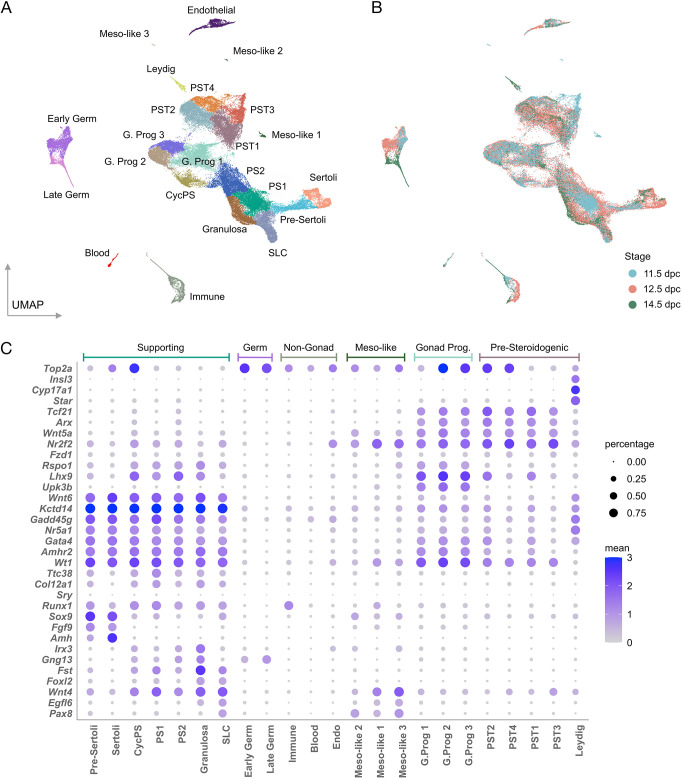
**scRNA-seq analyses of gonadal sex determination in C57BL/6J (B6J) wild-type and *Z*-del mouse embryos.** (A) UMAP showing ∼68,000 gonadal cells from three genotypes (XY WT, XX WT and XY *Z*-del) at three stages (11.5, 12.5 and 14.5 dpc). All major gonadal lineages are present. (B) UMAP of all cells coloured by stage (blue, 11.5 dpc; coral, 12.5 dpc; green,14.5 dpc). (C) Dot plots detailing expression specific to each cell type. Main supporting lineages express *Kctd14*; gonad progenitors express *Upk3b*; Leydig cells express *Star*; Sertoli cells express *Sox9* and *Amh*; SLCs express *Pax8*, although this is also found in other cell-types. CycPS, cycling pre-supporting cells; G. Prog, gonad progenitor; PS, pre-supporting; PST, pre-steroidogenic; SLC, supporting-like cells; Meso-like, Mesonephros-like.

Our previous studies of XY gonads lacking functional *Znrf3* took place on the sensitised C57BL/6J (B6J) genetic background (Bouma et al., 2007; Bogani et al., 2009; Harris et al., 2018). We characterised cellular differentiation in the fetal gonads of this sex-reversing mutant using scRNA-seq (see Materials and Methods for details of experimental design, methodologies and bioinformatic analyses). We examined the cellular composition of individual WT XY gonads (*n*=10), WT XX gonads (*n*=8) and XY *Z*-del gonads (*n*=17) throughout the sex-determining period (11.5, 12.5 and 14.5 dpc), as determined by unsupervised clustering of the transcriptomes of ∼68,000 cells, yielding the final all-cells Uniform Manifold Approximation and Projection (UMAP) (Becht et al., 2018) (Fig. 1A). This identified clusters corresponding to key gonadal cell lineages, including gonad progenitors, interstitial/pre-steroidogenic, Leydig, pre-supporting [three clusters, pre-supporting 1 (PS1), pre-supporting 2 (PS2) and cycling (dividing) pre-supporting (CycPS)], pre-Sertoli, Sertoli, granulosa (also known as pre-granulosa), germ cells and SLCs, a recently reported cell lineage found in mouse and human fetal gonads that contains *Pax8*-positive cells (Mayère et al., 2022; Garcia-Alonso et al., 2022). Sexual dimorphism is evident in this dataset, with pre-Sertoli (*Sox9*^+^, *Amh*^−^) and Sertoli (*Sox9*^+^, *Amh*^+^) cells and Leydig cells (*Star*^+^) detected exclusively in XY gonads, and granulosa cells (*Foxl2*^+^, *Fst*^+^, *Irx3*^+^) found only in XX (Fig. S1A,C). Moreover, contributions to these cell types are stage-dependent (Fig. 1A,B). For example, PS1 cells, representing bipotential supporting cell precursors that express *Gadd45g* (Warr et al., 2012; Stévant et al., 2019) and other genes associated with Sertoli and granulosa cell development (Fig. 1C), are found mainly at 11.5 dpc (Fig. 1B), whereas Sertoli and Leydig cells mostly appear at 12.5 dpc (Fig. 1B; Table S1). Dot plots (Fig. 1C) highlight expression of key markers in distinct cell lineages. Expression of *Gng13* at higher levels in PS2 suggests that some of these are granulosa cell precursors derived from the second wave of ovarian epithelial ingression (Zheng et al., 2014; Niu and Spradling, 2020). Fig. S1B also shows the expression of a number of key markers, many of which function in sex determination and differentiation, on the UMAP. Some of these, and additional markers, distinguish between pre-supporting cells in the developing XX and XY WT gonad (Fig. S2A,B). Many of these observations, which confirm those made in previous scRNA-seq studies of mouse sex determination (Stévant et al., 2018, 2019; Mayère et al., 2022), indicate a dataset that can be reliably interrogated to reveal the impact of loss of *Znrf3* on sex determination at the cellular level.

### Comparative analysis of B6J and CD1 gonads

**Fig. 2.**
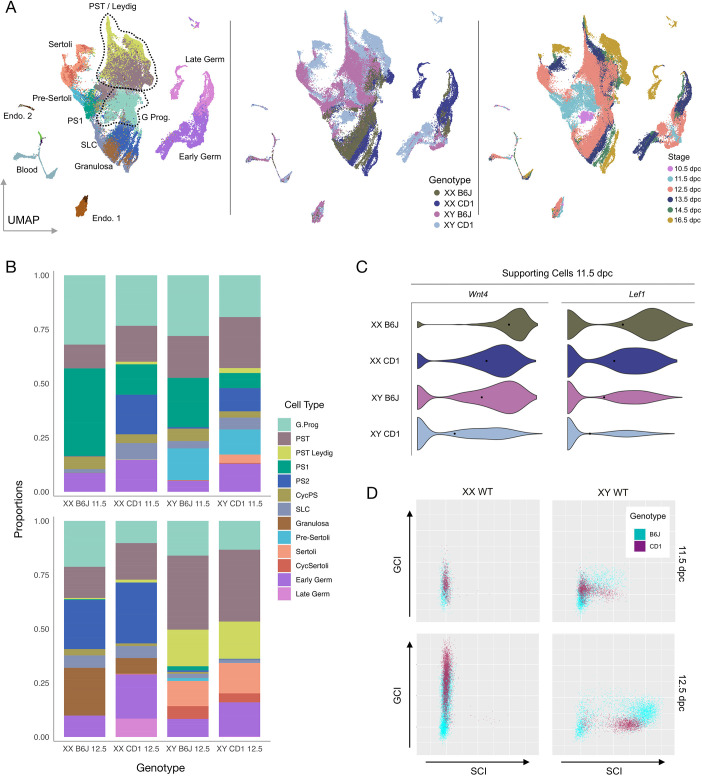
**Transcriptomic comparison of sex determination in the CD1 and B6J mouse strains.** (A) Combined CD1/B6J UMAP showing constituent cell types (left), strain of origin (middle) and stage of origin (right). The dotted lines group multiple sub-cell-types into one larger cell type. (B) Proportions of 13 different cell-types in B6J and CD1 datasets at 11.5 (upper) and 12.5 dpc (lower). (C) Violin plots showing expression of *Wnt4* and canonical WNT marker *Lef1* in the supporting cell lineage of the B6J and CD1 datasets at 11.5 dpc. (D) Plots of GCI (*y*-axis) and SCI (*x*-axis) scores found in cells from the B6J and CD1 supporting cell lineage datasets. Each dot represents an individual cell and its position on the SCI/GCI plot reflects its cellular identity regarding attainment of supporting cell (Sertoli or granulosa cell) fate. CycPS, cycling pre-supporting cells; GCI, granulosa cell identity; G.Prog, gonad progenitor; PS, pre-supporting cells; PST, pre-steroidogenic; SCI, Sertoli cell identity; SLC, supporting-like cells.

In order to allow an assessment of sex determination in the sensitised fetal B6J gonad at single-cell resolution, and provide a useful baseline for examining the gonadal sex reversal phenotype of *Znrf3* mutants on this background, we compared these data with a similar gonadal dataset generated in the 129/CD1 (referred to as CD1) strain (Mayère et al., 2022). CD1 is not a strain associated with sensitivity to disruption to testis determination; B6J and the closely related C57BL/6N are unique in this regard (Livermore et al., 2020). In order to compare the two strains, B6J was projected onto a CD1 dataset to obtain a combined UMAP (Fig. 2A). Then, using logistic regression, CD1 cells were associated with clusters previously described in B6J. There was good overlap between clusters representing those stages present in both datasets. Discordance was observed only for stages that are exclusively found in the CD1 dataset (10.5, 13.5 and 16.5 dpc) (Fig. 2A). We then compared the proportion of cells falling into 18 distinct clusters in the two strains in XY gonads at 11.5 and 12.5 dpc (Fig. 2B). Notably, at 11.5 dpc, the proportion of PS1 was significantly higher in B6J (*q*=4.69×10^−95^), suggesting a delay in commitment of supporting cell precursors to the Sertoli cell fate in this strain; by contrast, the proportion of Sertoli cells was higher in CD1 (*q*=8.49×10^−39^). In addition, analysis of canonical Wnt gene expression at 11.5 dpc in supporting cells showed significantly higher levels of *Wnt4* (*P*=2.15×10^−19^) and *Lef1* (*P*=2.71×10^−12^) in XY B6J compared with XY CD1 (Fig. 2C). Precocious and high *Wnt4* expression is thought to be a major contributor to the ‘feminised’ transcriptome of B6J gonads in the sex-determining period, which underlies its sensitivity to any further disruptions to testis determination (Munger et al., 2009).

In order to systematically analyse the sex determination process in the two strains as indicated by Sertoli cell and granulosa cell differentiation, we first identified transcriptomic signatures of Sertoli cell and granulosa cell differentiation by using machine learning to identify sets of genes (*n*=50) in the CD1 and B6J datasets (Table S2), expression (or absence) of which was most characteristic of WT Sertoli cell and granulosa cell development in each dataset. Based on similarity to this signature, a score for the degree of Sertoli cell or granulosa cell identity (SCI or GCI, respectively) was generated for each cell in the supporting lineage of each strain (see Materials and Methods for details). As expected, Sertoli and granulosa cell clusters in both datasets score highly with regard to SCI and GCI, respectively (Fig. S3A). We analysed the distribution of scores for GCI and SCI in the supporting lineage (including SLCs) of XX and XY WT CD1 and B6J gonads at 12.5 dpc (Fig. 2D). XX supporting cells had a similar distribution in both strains, apart from PS2, which was significantly diminished in B6J XX. The elevated number of granulosa cells as well as the diminishing number of PS2 cells in B6J XX compared with CD1 may concur with the feminisation of the B6J strain. XX supporting cells exhibited a continuous range of GCI scores, depending on degree of granulosa cell differentiation (Fig. S3B), with almost all cells clustered on the left-hand side of the graph, i.e. with little evidence of any significant SCI score for these cells in either strain. By contrast, analysis of XY cells in both strains revealed a discontinuous distribution of SCI scores evolving over time in both by 12.5 dpc (Fig. 2D), corresponding to whether supporting cells acquired Sertoli cell identity or not (Fig. S3B). Most cells in CD1 differentiate into Sertoli cells by 12.5 dpc. By contrast, a clear bimodal distribution is observed in B6J at the same stage, corresponding to high-SCI pre-Sertoli/Sertoli cells and a second group with lower SCI scores comprising all other supporting cell types (Fig. 2D; Fig. S3B). Notably, XY pre-Sertoli/Sertoli cells in B6J at 12.5 dpc had a significantly higher average GCI score than those in CD1 [mean B6J=26.91, *n*=2341; mean CD1=8.00, *n*=1363: *P*(mean)=0], with the range of GCI scores in XY B6J and CD1 pre-Sertoli/Sertoli cells also being significantly different [var(B6J Sertoli)=158.28; var(CD1 Sertoli)=37.30: *P*(var)=3.58×10^−164^]. Overall, this analysis supports the conclusion that gene expression and cellular composition in the B6J supporting cell lineage is ‘feminised’ in comparison with CD1, reflecting the sensitisation of B6J to disruptions to testis determination.

### Failure to commit to testis determination

**Fig. 3.**
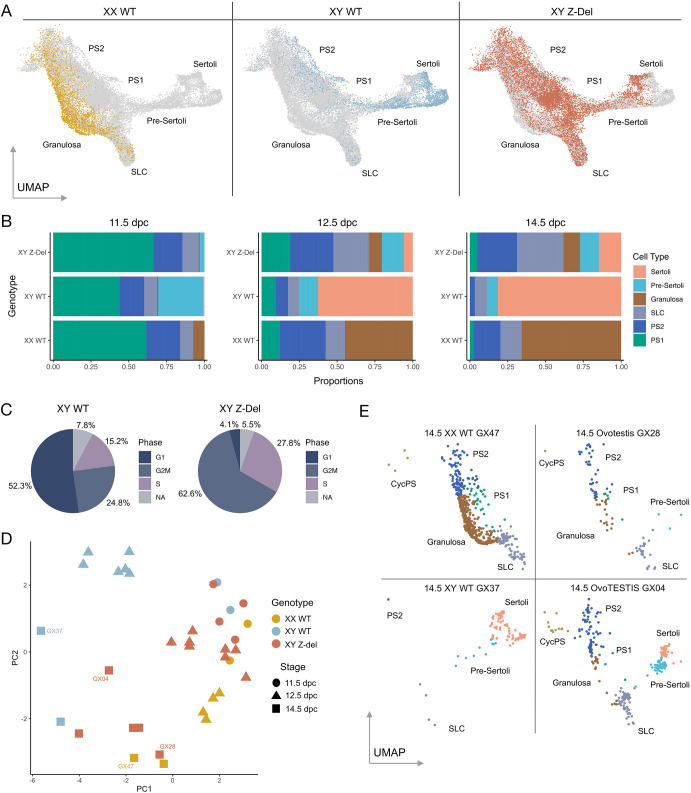
**The impact of loss of *Znrf3* function (*Z*-del) on gonadal cell transcriptomes in B6J.** (A) The supporting cell precursor and supporting cell regions of the all-cell UMAP, indicating the location of XX WT, XY WT and XY *Z*-del cells. (B) Proportions of supporting cell-types in the same three genotypes as A, at 11.5 dpc (left) 12.5 dpc (middle) and 14.5 dpc (right); cell-types are indicated by colour. (C) Pie-charts showing cell-cycle stage of Sertoli cells in XY WT and XY *Z*-del cells. (D) Principal component analysis of all samples analysed. (E) UMAPs derived from individual gonad samples at 14.5 dpc: XX WT (GX47), XY WT (GX37) and two XY *Z*-del samples; GX28 (an ovotestis) and GX04 (an ovoTESTIS). Cell-types are indicated by colour (key shown in panel B, right). CycPS, cycling pre-supporting; PS1, pre-supporting 1; PS2, pre-supporting 2; SLC, supporting-like cells.

We then performed a comparative analysis of B6J WT data and those from B6J XY *Z*-del mutants, focussing again on the supporting cell lineage, as this is the lineage in which the sex-determining ‘decision’ is first made, and this was our primary phenotypic interest in this mutant (Fig. 3). Examination of the distribution of cells of different genotypes across the supporting cell regions of the UMAP (Fig. 3A) shows, strikingly, that XY *Z*-del cells contribute to most supporting cell types, including pre-supporting, granulosa and pre-Sertoli/Sertoli. However, the proportion of XY *Z*-del cells in the granulosa cluster is relatively low in comparison with XX WT cells (Fig. 3B). Interestingly, XY *Z*-del Sertoli cells are only rarely (4.1%) found in the G1 cell-cycle phase (Fig. 3C) compared with XY WT Sertoli (52.3%). In contrast, *Z*-del pre-supporting clusters have similar distributions of cells in the G1 phase to XY WT, both >85% (Table S3). However, despite the majority of XY *Z*-del Sertoli at the later stages actively cycling, the XY *Z*-del cluster has a disproportionate reduction in Sertoli cells compared with XY WT (e.g. XY WT Sertoli is >60% of the supporting cell clusters, compared with ∼5% in the XY *Z*-del at 12.5 dpc). The lack of cells committing to the Sertoli cell fate in *Z*-dels, despite their cycling nature, remains unexplained, but could perhaps be reflecting the elevated WNT signals in the mutant (Figs S4, S5; Table S4). Differential expression analysis of Wnt4, known to be dysregulated in the absence of Znrf3 in gonad development (Harris et al., 2018), and LEF, a transcription factor WNT activates, are significantly highly expressed in XY *Z*-del compared with XY WT at all time points in supporting cells (Fig. S4). To learn more about the association of Znrf3 with the Wnt pathway we undertook differential expression analysis of supporting cells in XY *Z*-del versus XY WT, and 80 Wnt pathway genes (Kanehisa et al., 2023) were significantly dysregulated at 12.5 dpc (Table S4). In XY *Z*-del 47 Wnt genes were upregulated and 33 downregulated compared with XY WT. The top ten differential Wnt genes were examined further, in the supporting cells separately (Fig. S5), only two genes, *Fzd9* and *Plcb2*, showed XY lineage specific differences. These results show there is a plethora of significant canonical and non-canonical WNT pathway genes affected by *Znrf3* KO that warrant further analysis to elucidate their function.

These data reveal variability between XY *Z*-del mutants in respect of proportions of cells in the supporting lineage, as expected based on earlier studies of sex reversal in this mutant (Harris et al., 2018). A principal component analysis of these proportions (Fig. 3D; Table S5) shows that XY *Z*-del mutants at 12.5 dpc group with 11.5 dpc XX and XY WT samples, suggesting a failure of commitment to testis determination and differentiation. This analysis also indicates significant variation between XY *Z*-del samples at 14.5 dpc (Fig. 3D,E), with some aligning more closely with XX wild-type samples and others appearing more masculinised, in agreement with phenotypic annotation of the samples at dissection as ovotestis (GX28, Fig. 3D,E) or ovoTESTIS, referring to samples with increased testicular tissue formation (GX04, Fig. 3D,E). At this stage, XY WT cells are found primarily in the Sertoli cell and pre-Sertoli clusters but not in pre-supporting clusters (GX37, Fig. 3E). By contrast, XY *Z*-del cells are found in pre-supporting clusters (PS1 and PS2) as well as the granulosa cluster (GX28 and GX04, Fig. 3E). This increase in the numbers of pre-supporting cells in the *Z*-del mutants (e.g. PS1, q=1.25×10^−33^) suggests a failure of commitment to a differentiated supporting cell fate, i.e. Sertoli or granulosa cell.

One other notable feature of the XY *Z*-del gonads was a greatly increased proportion of cells in the SLC cluster when compared with XY WT (Fig. 3B,E; Table S5). As increased numbers of SLCs would be predicted to be associated with elevated *Pax8* expression, we used RNAscope to detect *Pax8* on gonadal sections at 12.5 dpc, also using *Nr5a1* as a gonadal somatic cell marker (Fig. S6). In addition to prominent expression in the mesonephric tubules, *Pax8* is found in WT gonadal tissue primarily at the junction with the mesonephros at 12.5 dpc, with expression also in the area of the rete testis (Fig. S6A), in agreement with Mayère et al. (2022). In XY *Z*-del tissue, increased *Pax8* expression was detected at the poles of ovotestes, where ovarian tissue is more common (Fig. S6B,C). However, it is not possible to unambiguously attribute all of this *Pax8* expression to the presence of SLCs, given expression of *Pax8* in other gonadal cell-types (Fig. 1C). Increased *Pax8* expression in ovarian regions of the XY mutant gonad led us to hypothesise that ovarian tissue lacking ZNRF3 exhibits particularly high levels of *Pax8* expression. We tested this by examining *Pax8* expression in WT XX and *Z*-del mutant XX gonads. As predicted, when compared with XX WT (Fig. S6D), higher levels of *Pax8* expression were observed in the XX Z-del mutant tissue along the length of the gonad, with highest levels still observed closer to the mesonephros (Fig. S6E,F). SLCs are thought to contribute to the rete testis and ovarii and we have shown that expression of *Sox9* marks the region of the rete in XY and XX gonads following wholemount *in situ* hybridisation (WMISH) (Mayère et al., 2022). No overt abnormalities of *Sox9* expression in the rete region of the gonad/mesonephros were observed in XY *Z*-del embryos at 13.5 dpc (Fig. S6G).

**Fig. 4.**
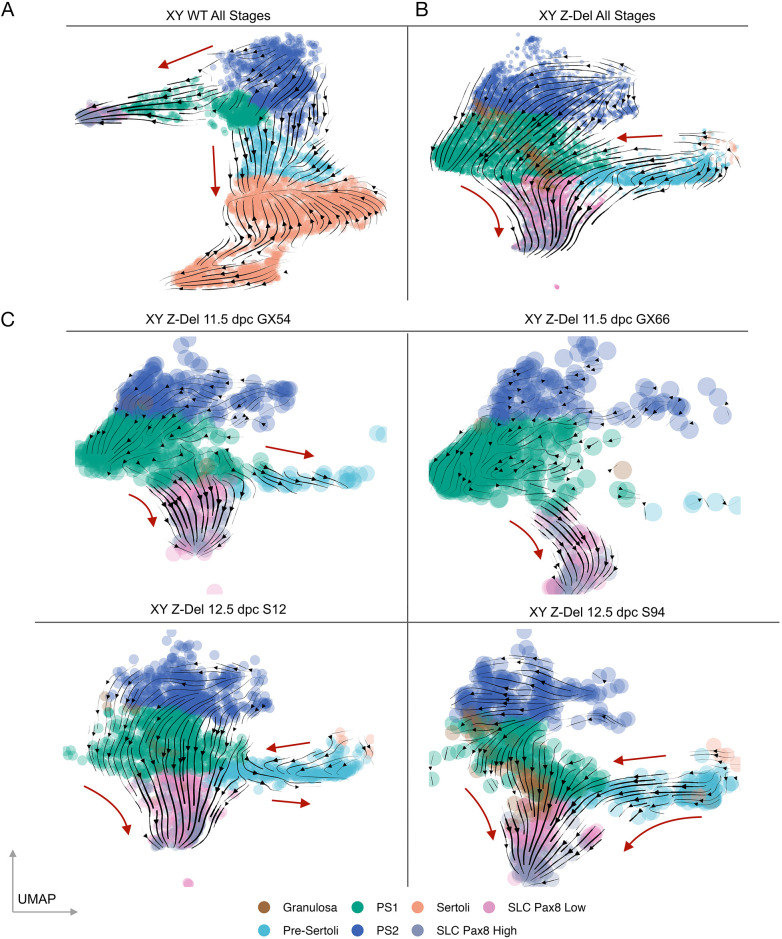
**Predicted alteration to cellular differentiation trajectories in B6J XY *Z*-del gonads.** (A-C) RNA velocity trajectories (arrowheads on black lines) are shown on UMAPs from XY WT, which combines samples from all stages (A), XY *Z*-del samples from all stages (B) and individual XY *Z*-del samples at 11.5 and 12.5 dpc (C). The red arrows further indicate the general direction of the individual trajectories. For XY *Z*-del, in the absence of a Sertoli cluster, the predicted trajectory is towards pre-supporting and SLC cell-types. PS1, pre-supporting 1; PS2, pre-supporting 2; SLC, supporting-like cells.

We then analysed cellular developmental trajectories in the supporting cell lineage of XY WT control and XY *Z*-del mutants using RNA velocity (La Manno et al., 2018), which examines the ratio of spliced to unspliced forms of each transcript to determine the direction of transcriptional modulation and extrapolate developmental fate (Fig. 4). When all stages of XY WT gonads are analysed, they are characterised by an overall trajectory from supporting cell precursors (PS1/PS2) towards pre-Sertoli and, ultimately, Sertoli cells (Fig. 4A). A trajectory from PS1 towards SLCs is also observed (Fig. 4A). When all XY *Z*-del gonadal cells are analysed (Fig. 4B), there is a consensus trajectory of PS1 cells, which exist in larger numbers when compared with XY WT (Fig. 3B), towards SLCs (Fig. 4B,C). There is also an apparent gradient in the levels of *Pax8* expression in the SLC cluster; those showing lower *Pax8* expression cluster more closely to granulosa cells in the UMAP and it is these ‘*Pax8*-low’ SLCs that are found in especially high numbers in XY *Z*-del gonads. Variability in altered *Z*-del cellular trajectories is noticeable when individual mutant gonad samples are analysed (see Fig. 4C, where four independent *Z*-del samples are shown at two stages). A trajectory in one mutant from PS1 to XY granulosa is observed (S94, Fig. 4C), as well as a trajectory from XY granulosa cells to SLCs (same sample).

In XY *Z*-del gonads, there is also much more limited contribution to pre-Sertoli cells (see 11.5 dpc samples GX54/GX66, Fig. 4B). Interestingly, at 12.5 dpc there is a clear trajectory away from pre-Sertoli/Sertoli cell fate, towards *Pax8*-low SLCs (Fig. 4B). Overall, these results of *Z*-del gonad analysis are notable for the following features: (1) accumulation of cells on the pre-supporting path (PS1) that have failed to commit to the pre-Sertoli path, and the transition of a proportion of these to granulosa cell fate; (2) cells that have committed to the Sertoli path but exhibit a trajectory back towards SLC fate; (3) large numbers (larger than in WT) of pre-supporting cells also committing to the SLC fate, especially the *Pax8*-low type. A proportion of these also transition to *Pax8*-high SLCs (Fig. 4). This is consistent with elevated expression of *Pax8* in XY and XX *Z*-del gonads observed by RNAscope described above.

### Pervasive disruption to supporting cells

**Fig. 5.**
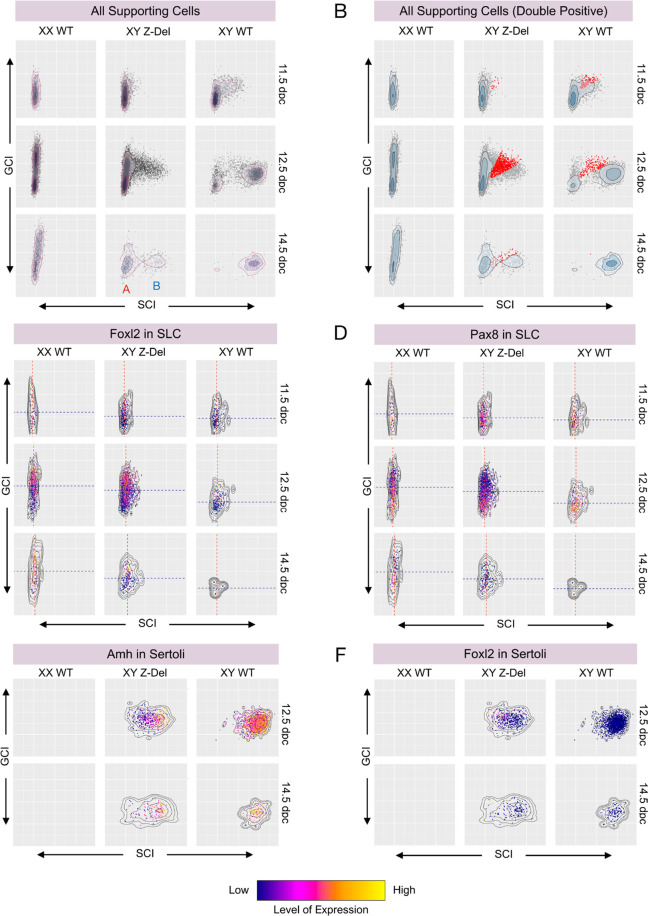
**Pervasive disruption to attainment of cellular sex identity in B6J XY *Z*-del gonads.** (A) Density scatter-plots of GCI and SCI scores for all supporting cells in XX WT, XY *Z*-del and XY WT gonads. Density scatter plots chart the variation in SCI (*x*-axis) and GCI (*y*-axis) scores in these three genotypes across three stages – 11.5 dpc (top), 12.5 dpc (middle) and 14.5 dpc (lower) – in a 3×3 grid. Each dot represents an individual cell and its identity regarding attainment of supporting cell (Sertoli and granulosa cell) fate. For XY *Z*-del at 14.5 dpc, ‘A’ refers to a low SCI score group, corresponding to supporting cells that are not pre-Sertoli/Sertoli; ‘B’ refers to a high SCI score group comprising pre-Sertoli/Sertoli cells. (B) GCI/SCI plot for all supporting cells, showing cells with similar GCI and SCI scores (‘double-positive’, DPs) indicated in red. At 12.5 dpc and 14.5 dpc, the proportion of DPs in *Z*-dels has increased compared with XY WT. (C) GCI/SCI plot for supporting-like cells (SLC), with *Foxl2* expression levels indicated by colour. The average SCI and GCI score for SLCs is indicated by a red and blue trend line, respectively. SLCs have increased *Foxl2* expression and higher than average GCI in *Z-*dels when compared with XY WTs. (D) GCI/SCI plot for SLCs, with *Pax8* expression levels indicated by colour. Trend lines shown are as in C. SLCs have decreased *Pax8* expression in cells with high GCI. (E) GCI/SCI density scatter plot for Sertoli cells, with *Amh* expression levels indicated by colour. In XY *Z*-del gonads, Sertoli cells have a reduced SCI score, with significantly lower *Amh* expression when compared with XY WT, at 12.5 dpc. (F) GCI/SCI plot for Sertoli cells, with *Foxl2* expression levels indicated by colour. In XY *Z*-del gonads, Sertoli cells have a reduced SCI score, with significantly higher *Foxl2* expression compared with XY WT, at 12.5 dpc. GCI, granulosa cell identity; SCI, Sertoli cell identity.

Finally, we used a whole transcriptome approach to examine the acquisition of Sertoli cell or granulosa cell fate in WT and mutant gonads by again using the SCI/GCI scoring method described earlier when comparing CD1 and B6J. A density scatter plot of SCI and GCI scores of all cells from the supporting lineage in control gonads revealed progressive attainment of higher GCI and SCI scores in XX and XY WT B6J gonads, respectively, over the three time-points analysed, most notably for XY, which culminates with the vast majority acquiring an *Amh*^+^, *Foxl2*^−^ Sertoli fate (Fig. 5A; Fig. S7A,B). A plot for XY *Z*-del mutant gonads reveals a major disruption to the attainment of SCI scores at WT levels. *Z*-del supporting cells attain a bimodal distribution, consisting mostly of group A and B cells (Fig. 5A). Group A cells have low SCI scores; this group of cells is essentially absent from XY WT gonads at the same stage. A second group of cells (group B) exists with higher SCI scores. Analysis of the cell-type composition of these two major groups reveals that group B corresponds to pre-Sertoli/Sertoli cells (Fig. S7C); group A contains pre-supporting cells, granulosa cells and SLCs (Fig. S7C). This observation is consistent with the trajectory analysis above.

We then addressed the identity of cells in the XY *Z*-del mutant that appear to fall between these two groups of supporting cells described above, both at 12.5 and 14.5 dpc. Given that these cells have a SCI score that is intermediate between the Sertoli group (B) and all other supporting cells (A), we reasoned that this would be due to reduced expression of genes in the Sertoli cell signature and, possibly, elevated expression of some genes in the granulosa cell signature. To identify cells with significant co-expression of genes in the two expression signatures, we highlighted all cells with a similar GCI and SCI score (cells coloured red, Fig. 5B and Materials and Methods). Such cells, which we termed ‘double-positives’, are detected in both B6J XY WT and XY *Z*-del mutant gonads, although are present at negligible levels by 14.5 dpc in XY WT (Fig. 5B). Double-positive cells are primarily detected in WT gonads at 12.5 dpc in the PS1/2, pre-Sertoli and SLC clusters (Table S6). Notably, the proportion of double-positive cells in the XY *Z*-del supporting lineage is higher than in XY WT at 12.5 dpc (*P*=1.82×10^−38^) (Fig. 5B; Table S6). In CD1, at 11.5 and 12.5 dpc, double-positive cells are uncommon, in contrast to the situation in XY B6J WT and XY *Z*-del, highlighting again the relative rapidity of the commitment to testicular (Sertoli) cell fate in CD1 (Fig. S8A). In the XY *Z*-del mutant, double-positive cells are found in the same clusters as in WT gonads (Table S6), but with significantly higher contributions from particular cell types, namely PS1, SLC, pre-Sertoli and Sertoli cells (Fig. S8B-E). We confirmed the prominence of double-positive cells at 12.5 dpc in *Z-*del mutant gonads by examining expression of specific gene-pairs (one pro-testis, one pro-ovary) in control and mutant gonads, again using RNAscope (Figs S9 and S10). We analysed one gene pair based on the literature of sexually dimorphic genes (*Sox9*/*Fst*; Fig. S9) and one pair derived from our analysis of the Sertoli cell and granulosa cell signatures identified in this manuscript (*Col18a1*/*Sulf1*; Fig. S10). This revealed clear co-expression of such gene pairs in the XY *Z*-del mutant at levels not observed in XY or XX control gonads, in which expression was sex-specific at this stage, consistent with the scRNA-seq data (Fig. S11).

We further analysed the distribution of SCI and GCI scores in two cell-types, SLCs and Sertoli cells, in order to shed light on the disruption to the transcriptomes of these cell-types in mutant gonads (Fig. 5C-F; Table S7). SLCs in XX WT gonads at 12.5 dpc were characterised by prominent *Foxl2* expression in those cells with the highest GCI score (Fig. 5C). Interestingly, this coincided with lower overall *Pax8* expression (*q=*2.81×10^−37^); however, SLCs with lower GCI scores exhibited higher *Pax8* expression (Fig. 5D), in a pattern that appears to be reciprocal to that of *Foxl2*. This pattern was preserved in *Z*-del mutants at the same stage, in contrast to XY WT controls, which exhibited fewer SLCs (*q=*2.14×10^−83^), lower GCI scores (*q*=2.16×10^−59^) and lower *Foxl2* expression (*q*=9×10^−18^) (Fig. 5C,D). These data, alongside the 14.5 dpc results (Tables S7 and S8), indicate a shift in the SLC transcriptome of XY mutant cells towards that found in XX WT cells i.e. the ovarian fate, a form of gonadal sex reversal.

When cells from the Sertoli cell cluster were analysed in respect of their SCI/GCI scores at 12.5 (and 14.5 dpc), the reduction in SCI scores in the *Z*-del mutant (*q*=3.41×10^−175^) coincided with a reduction in *Amh* expression (*q*=5.23×10^−85^) and the increase of cells expressing *Foxl2* (*q*=2.21×d10^−20^) (Fig. 5E,F; Tables S7 and S8). Overall, our analyses of SCI and GCI scores in supporting cells of our dataset reveals a pervasive disruption to the attainment of sex identity in mutant gonads, which extends even to cells that are found in the Sertoli cell cluster, many of which exhibit a shift towards a less robustly testicular transcriptome and might best be described as Sertoli-like. Our analyses reveal that few supporting cells appear to escape the impact of *Znrf3* mutation.

## DISCUSSION

The data reported here comprise a single-cell transcriptional atlas of gonadal sex determination in the most commonly used strain, C57B6L/6J (B6J). Investigations of testis determination are especially fruitful in B6J, as it is a strain that is sensitised to disruptions to testis development and XY gonadal sex reversal phenotypes are therefore more common on this background (Eicher et al., 1982; Munger et al., 2009). The transcriptomic and cellular basis of this sensitisation are revealed in our dataset when it is compared with a similar dataset generated in CD1 mice (Mayère et al., 2022). Higher proportions of pre-supporting cells are present in XY B6J gonads, indicating a more rapid transition in CD1 towards Sertoli cell fate. Moreover, by using transcriptomic signatures of Sertoli cell and granulosa cell identity, we show that alongside the heightened expression of pro-granulosa genes in B6J, there are higher proportions of cells also expressing both pro-Sertoli and pro-granulosa genes, consistent with earlier reports of the ‘feminisation’ of this genetic background.

Using the same approach, of calculating a score for the attainment of Sertoli (SCI) or granulosa (GCI) cell fate, alongside an analysis of cellular trajectories in control and mutant gonads, we show that fetal gonadal sex reversal in *Z*-del mice is characterised by the pervasive disruption to cell fate acquisition. The proportions of different gonadal cell-types are altered in XY *Z*-del mutant gonads, with fewer Sertoli cells, increased levels of supporting cell precursors (PS1 cluster) at all stages, XY granulosa cells developing and larger numbers of supporting-like cells (SLCs). These altered proportions are not consistent with the cycling properties of the XY *Z*-del Sertoli cluster, which is actively proliferating but not significantly increasing in proportion. These altered proportions are however consistent with an analysis of cell trajectories using RNA velocity, indicating future fates not reflecting robust Sertoli cell differentiation, but instead a form of ‘backing-up’ of supporting cell precursors, trajectories towards SLCs and, perhaps most curiously, away from pre-Sertoli/Sertoli cells. Analysis of the transcriptomes of supporting cells using the SCI/GCI scoring method reveals widespread disruption in mutant gonads, even extending to the transcriptomes of cells that have achieved Sertoli cell status, i.e. are in the Sertoli cell cluster of the all-cell UMAP, which have shifted to varying degrees away from the core Sertoli cell transcriptomic signature towards a more pro-granulosa cell signature. The functional consequences of such shifts in a cell that is still identified as a Sertoli cell remain unclear.

The disruption to transcriptional fate in mutants includes the accumulation of large numbers of ‘double-positive’ cells, characterised by the expression of Sertoli and granulosa cell pathway (signature) genes, including at 14.5 dpc when these are absent from WT controls. Such cells are detectable in greatly reduced numbers at 12.5 dpc in XY WT controls and represent bipotential precursors that, at least in B6J gonads, have yet to transition to the pre-Sertoli/Sertoli path. It is commonly thought that, on the basis of the mutual antagonism that occurs between the testis- and ovary-determining pathways, their co-existence is exceedingly rare, even in pathogenic circumstances. For example, immunohistochemical analyses of SOX9 and FOXL2, which are both detected in ovotestes at the tissue level, reveals that they are only very rarely co-expressed at the cellular level, both in mice (Wilhelm et al., 2009) and humans (Hersmus et al., 2008). Our data suggest that this might represent a feature of those two particular transcription factors, perhaps based on their shared chromatin associations (Rahmoun et al., 2017; Nicol et al., 2018), but is nevertheless not a feature that can simply be extrapolated to all genes in the pro-testis and pro-ovary genetic pathways. Further analysis of this unusual cellular co-expression of pro-testis/pro-ovary genes is warranted in gonads in different genetic contexts in mice and humans, including in individuals with disorders/differences of sex development (DSD).

In addition to disruption to cells of the supporting cell lineage that contribute to the pool of Sertoli and granulosa cells in the developing gonad, in which the sex-determining signal is first initiated, we also report disruption to the transcriptomes of SLCs in XY mutant gonads. XY SLCs lacking *Znrf3* acquire a transcriptome closely resembling that of XX SLCs, with prominent expression of genes also associated with granulosa cells, such as *Foxl2*. This transcriptomic feminisation suggests that the sex reversal caused by loss of ZNRF3 extends beyond the supporting cell lineage proper, impacting SLCs too. Moreover, the numbers of SLCs in XY *Z*-del mutant gonads also increases. In particular, we observe transition of supporting cell precursors and pre-Sertoli cells towards a pre-supporting cell fate as well as a *Pax8*-low SLC type, which themselves transition to *Pax8*-high SLCs. This increase in SLC numbers is consistent with the increase in gonadal expression of *Pax8* detected in mutants. Indeed, this increase is clearly observable in XX *Z*-del mutants too. These data support a role for WNT signalling in promoting SLC development. This conclusion is consistent with a recent report that loss of *Wnt4* in XY gonads negatively impacts SLC development, resulting in fewer *Pax8*-positive cells and disruption to formation of the rete testis (Mayère et al., 2022). We did not observe overt abnormalities of the rete in mutants, which suggests that the ‘excess’ SLCs of *Z*-del mutants may instead occupy other regions of the gonad, consistent with elevated *Pax8* expression in *Znrf3*-deficient ovarian tissue. These observations raise questions concerning the role of SLCs that remain to be answered. Future studies will need to address whether some SLCs, perhaps those most proximal to granulosa cells transcriptomically, might contribute to the granulosa cell pool, i.e. follicles, in certain circumstances, as recently postulated (Mayère et al., 2022). A marker which is unique for SLCs and which does not detect granulosa cells would help in this respect. Indeed, our own previous report of ectopic *Foxl2* and *Wnt4* expression XY *Z*-del gonads, which was attributed to the appearance of granulosa cells (Harris et al., 2018), can now be attributed to a combination of XY granulosa cell and enhanced numbers of ‘feminised’ SLCs.

Mayère et al. (2022) describe an origin for SLCs in an early multipotent progenitor population of the newly formed mouse gonad. Here, we report a contribution to the SLC lineage from supporting cell precursors and even granulosa cells in WT XY and XX gonads, respectively, which is enhanced by mutation of *Znrf3*. The data reported here did not include samples from 10.5 dpc, and thus the question of a similar early origin for SLCs in B6J gonads cannot be readily addressed. But the observations of Mayère et al. in CD1 and our data from B6J are not irreconcilable, as SLCs may have complex origins and, indeed, a complex set of fates, given that we have described heterogeneity in this cell-type based on *Pax8* expression alone. Further research is required to fully understand this newly described cell-type and all its roles in gonad development and adult gonad function.

Gonadal sex reversal is often associated with a relatively simplistic model in which an ovary forms in an XY fetus or, much more rarely, a testis forms in an XX individual. Indeed, there do appear to be some examples of relatively ‘pure’ gonadal sex reversal. Our previous analyses of sex reversal in mice lacking GADD45γ, for example, indicate the formation of ovarian tissue throughout the whole of the developing gonad without any obvious delay in the timing of various key molecular or cellular events (Warr et al., 2012). Other studies of abnormal sex determination in mice have revealed disruptions to a variety of processes that result in gonads with an identity that is more difficult to describe in simple terms. For example, loss of pro-ovarian *Rspo1* in XX gonads results in a degree of masculinisation of the fetal gonad, with the appearance of a coelomic vessel and steroidogenic cells, but the absence of testis cord formation (Chassot et al., 2008). In any given genetic background, it has been clear for many years that it is not simply a question of whether a testis or ovary forms. Natural genetic variation, transgenesis or loss-of-function mutation, and combinations of these, can disrupt this binary outcome and lead, for example, to ovotestis development (Eicher et al., 1982; Warr et al., 2014; Livermore et al., 2020). Our studies of cellular differentiation in the XY *Znrf3*-deficient mutant gonad suggest that the non-binary model should be extended to sexual differentiation of individual cells of the supporting cell lineage: it is not simply a case of Sertoli or granulosa cell differentiation, but the degree to which differentiation of these is achieved. Our report of pervasive disruption to the attainment of sex identity in the XY *Znrf3* mutant, including the appearance of Sertoli cells with atypical, ‘feminised’ transcriptomes, which might be called ‘Sertoli-like cells’, may prompt reassessment of the cellular phenotypes associated with disruption to sex determination in other genetic contexts. As opposed to the familiar use of one or two cellular markers to analyse each gonadal cell lineage in a mutant, which may mislead, we propose introducing scRNA-seq as a standard phenotyping tool that will avoid any misattribution of cell-type status to cells in atypical genetic contexts and reveal disruptions to cellular and molecular events in sex determination in a much more granular and therefore reliable fashion.

## MATERIALS AND METHODS

### Mouse strains and embryo collection

*Znrf3* mutant mice, previously described (Koo et al., 2012; Harris et al., 2018), were maintained on the C57BL/6J (B6J) background. All animal experiments were approved by the Animal Welfare and Ethical Review Body at MRC Harwell Institute. Mice used were bred under license from the UK Home Office (PPL 70/8898 and PP5230673). Mice were housed in individually ventilated cages in a specific pathogen-free environment. Further details of micro- and macroenvironmental conditions are available on request.

Noon on the day that the copulatory plug was detected was counted as 0.5 dpc. Mice were killed by dislocation of the neck, which was confirmed by palpation, and then embryos were decapitated in ice-cold, phosphate-buffered saline solution. Embryos collected at 11.5 and 12.5 dpc were staged accurately based on the number of tail somites (ts), here 18-19 ts (11.5 dpc) and 25-32 ts (12.5 dpc). Gonads at all three stages were carefully dissected away from the mesonephros.

### Cell capture, library synthesis and sequencing

Dissected gonads were dissociated in trypsin for 10 to 15 min, at 37°C, depending on stage. The reaction was quenched with fetal bovine serum and, following centrifugation (250 ***g***), cells were resuspended in phosphate-buffered saline/0.4% bovine serum albumin. Cell viability was assessed with Trypan Blue and haemocytometer analysis. Most gonads were then immediately fixed with 100% ice-cold methanol to allow sample preservation before cell capture (method modified from Alles et al., 2017) (Table S1). The fixation had no adverse effects on the samples: both fixed and unfixed cells at 12.5 dpc fully integrate on the UMAP (Fig. S12). Fixed and unfixed 12.5 dpc gonads were used in subsequent analyses. All 11.5 and 14.5 dpc gonads were fixed (Table S1).

Samples were transported to the Oxford Genomics Centre, Wellcome Centre for Human Genetics (Oxford, UK), for cell capture on the 10x Chromium System. scRNA-seq libraries were generated using the Chromium Single Cell 3′ v.2 assay (10x Genomics). Libraries were sequenced using the X platform (Illumina) to a depth of ∼50,000 reads per cell. Raw reads were aligned to the mouse genome (mm10) and cells were called using Cell Ranger count (v.4.0.0).

### Filtering of low-quality cells

To identify doublets, Scrublet (v.0.2.1) was used with default parameters; cells annotated as doublets were removed from the dataset. Seurat (v.3.2.2) was used to remove low quality cells based on low numbers of genes expressed, counts and abnormal percentages of mitochondrial RNA, where each acts as a potential indicator of cell death or rupture. Cells falling outside quality control thresholds were removed from the dataset first on a per sample basis and then for the whole dataset. For each sample, cells with UMI counts of <2% and >98% of the total were removed, genes expressed in <2% and >98% of the total were removed and mitochondrial RNA percentage expressed in <2% and >95% of the total were removed. However, low quality cells were still present, therefore a global filter was applied to the whole dataset to exclude cells in the bottom 5th percentile of counts, i.e. cells with less than ∼5000 counts.

### Cell cycle assignment

Seurat (v.3.2.2) was used to identify cell cycle phases in each sample using the cell cycle scoring function CellCycleScoring. Mouse genes corresponding to cell cycle phases G2/M and S were downloaded from Ensembl v98.

### Cell clustering and cell-type annotation

Monocle 3 was used to identify clusters of cells. Cells were prepared using the pre_process_cds function and batch correction was performed using mutual nearest neighbours via the align_cds function, with ‘sample’ specified as the means by which to integrate. Cells were clustered using the graph-based Leiden community detection algorithm. The top_marker function was used to find genes that are highly expressed and specific to each cell cluster. Cells were automatically labelled with the top two specific genes. Cell types were labelled using known markers of gonadal cell types. Clusters were then visualised via UMAP. Three different types of UMAP were generated: an all-cells UMAP consisting of all XY WT, XX WT and XY *Z*-del samples, as well as a XY WT-only UMAP and a XY *Z*-del-only UMAP.

### Statistical analysis

To assess the variation in different cell types in all the samples, a principal component analysis was performed on the proportions of all gonadal cell types for all samples (excluding blood, immune, mesothelial and endothelial clusters).

All cell proportion differences were tested using the chi-square method. Differential expression was calculated by regression analysis in Monocle 3 and genes classified as differential where q-value <0.05. The magnitude and direction of the differentials is described by the estimate.

### Comparative analysis of CD1 and B6J scRNA-seq datasets

Count data from Mayère et al. (2022) containing 129/CD1 cells along XX and XY mouse gonadal development were aggregated with data from the B6J XX and XY dataset based on the intersection of genes present in both datasets. Aggregated counts were normalised to a target sum of 10,000 counts per cells, excluding the top 5% highly expressed counts, and log transformed.

In order to project B6J data on the CD1 atlas, dimensionality reduction of B6J data was predicted through the Independent Component Analysis model (ICA) trained on the CD1 data. Data was centred on the ICA model centre, and multiplied by the whitening matrix, transposed, and multiplied by the orthogonal rotation matrix to obtain the components according to the ICA loading obtained using CD1 data. Then to increase data overlap, a neighbour graph was computed using the BBKNN (Polański et al., 2020) package on the ICA matrix, using batch as a covariate. The batch covariant categories were sequencing batch and sequencing lane for CD1, and sample-wise for BJ6.

A UMAP was generated using the combined B6J and CD1 ICA data as input, via Scanpy's UMAP function with min_dist parameter at 0.2. Independently, to find correspondence between cell types in the two datasets, a logistic regression classifier (Pedregosa et al., 2011) model was trained on a subset of the B6J transcriptome. The subset consisted of 500 randomly picked cells per cell type in B6J data. The cell-type classification of the cells from the CD1 atlas were then predicted with the classifier.

### RNA velocity analysis

Velocyto (v.0.17.17) was used to generate loom files to store calculated velocity data. ScanPy (v.1.6.0) was used in conjunction with SCVelo (v.0.2.2) to prepare and process the scRNA-seq samples. Velocities were assessed using the stochastic model with default settings. The cluster annotations and UMAP coordinates of the XY WT and XY *Z*-del UMAPs were used for velocity analyses. Cells were graphed using a ScanPy version of the UMAPs and summaries of cell velocities are represented by the summary arrows on the image.

### Identifying gonadal cell identity

We used a non-biased machine learning approach to determine the tendency of a cell to develop into a granulosa or Sertoli cell. Using the summary of gene expression, two separate models were used to generate a ‘granulosa-ness’ and ‘Sertoli-ness’ score for each cell, namely, a ‘granulosa cell identity’ (GCI) and a ‘Sertoli cell identity’ (SCI) score, respectively. These scores are calculated for every cell and allow us to determine the attainment of Sertoli or granulosa cell fate, or intermediate fates.

### Gene assessment

To increase the accuracy of GCI and SCI scores, we used a targeted approach to rank the genes as candidates for use in a machine learning model, rather than use the most variable genes. For granulosa cells, each expressed gene was assessed for its ability to discriminate between granulosa cells and other cell types within the XX sample. The same method was used for Sertoli cells and the XY samples. This identifies pro-granulosa cell markers and pro-Sertoli cell markers by giving them a score to rank their association with cell-type differentiation. Specifically, we created a generalised linear model (GLM) using gene expression only, with the area under the receiver operating characteristic (ROC) curve (AUC) used as a metric to generate a ranked list of genes as input to the machine learning algorithm. All genes were given a score for both cell types, and genes might occur predominantly in both lists as a positive or negative indicator. For example, a gene may be a positive indicator in Sertoli cells and a negative indicator in granulosa cells, or vice-versa. Alternatively, a gene may conceivably be a positive discriminator in both lists if it discriminates both granulosa cells and Sertoli cells from the other cell types.

### Model building

Using a GLM-based model, the ranked genes were sequentially added to the model and the results assessed using cross-validation. If a gene did not add to the model cell type discrimination sensitivity (via ROC AUC), the gene was excluded in a method analogous to a stepwise AIC method. When the model reached a maximum discrimination, the addition of genes was stopped. Additional gain of discrimination ability for granulosa-ness and Sertoli-ness was reached at ∼25 genes and ∼50 genes, respectively; therefore, the final model used 50 genes for each score, to allow consistency between models.

A model was then fitted for each of the two gene sets using Sertoli cells and granulosa cells from each mouse strain as the training data. These models can then be used to predict granulosa and Sertoli cell types in other samples, which yields a score for transcriptomic similarity to the desired cell type. This method produced one score for Sertoli cell discrimination and a second score for granulosa cells, the SCI and the GCI. For example, Sertoli cells have a high SCI score, and granulosa cells a high GCI score. By calculating these scores for every cell, we can plot each cell in a two-dimensional CIS-space graph, plotting the GCI score for each cell versus its SCI score. The unpaired two-sample Wilcoxon test was used to determine significant differences in SCI and GCI between XY *Z*-del and XY WT.

### Identification of double positive cells

Double positive cells have similar SCI and GCI scores. To identify cells with similar SCI and GCI scores, we defined a hyperbola using the equation below, where a is a scaling factor of 10 and b is the threshold or boundary of the hyperbola, represented by the 97.5% percentile of GCI scores in 14.5 dpc XX Granulosa WT cells. This returns TRUE if the cell is within the defined region. These cells are called double positive cells.

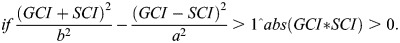
To compare the proportions of double positive cells with non-double positive cells, we used a chi-squared test (with a false discovery rate).

### Tissue expression studies

RNA *in situ* hybridisation using RNAscope Multiplex Fluorescent V2 Assay was performed according to the manufacturer's instructions using probes for *Pax8*, *Nr5a1*, *Sox9*, *Fst*, *Col18a1* and *Sulf1* (Advanced Cell Diagnostics). WMISH with *Sox9* has been previously described (Bogani et al., 2009). For image processing and adding scale bars, ImageJ was used (Fiji; https://imagej.net/software/fiji/).

### WNT signalling pathway genes

WNT genes were annotated based on the KEGG WNT Signalling Pathway downloaded from the KEGG Database (Kanehisa et al., 2023).

## Supplementary Material



10.1242/develop.202707_sup1 Supplementary information

Table S1. Proportions of distinct cell-types in XY Z-del mutant and B6J control (XX and XY wild-type (WT)) gonads, per sample, at all stages.

Table S2. Gene expression signatures defining Sertoli cell and granulosa cell differentiation in B6J and CD1 WT gonads.

Table S3. Number of cells per cycle phase at each time point in each supporting cell type cluster in B6J XY Z-del, B6J XY WT, and B6J XX WT.

Table S4. Differential gene expression of classical Wnt genes between B6J XY Z-del and B6J XY WT.

Table S6. Proportions of double-positive cells in XY Z-del mutant and B6J control (XX and XY wild-type (WT)) gonads at all stages in distinct cell-types.

Table S7. Average SCI/GCI scores and significance in supporting cell-types in XY Z- del mutant and B6J XY wild-type, at 12.5 dpc and 14.5 dpc.
