# Supplementary material for: Gonadal sex reversal at single-cell resolution in Znrf3-deficient mice
Source: Development. 2024 Dec 4;151(23):dev202707. doi: 10.1242/dev.202707 (PMC11658683; doi:10.1242/dev.202707)
Supplement: Supplementary information [file develop-151-202707-s1.pdf]

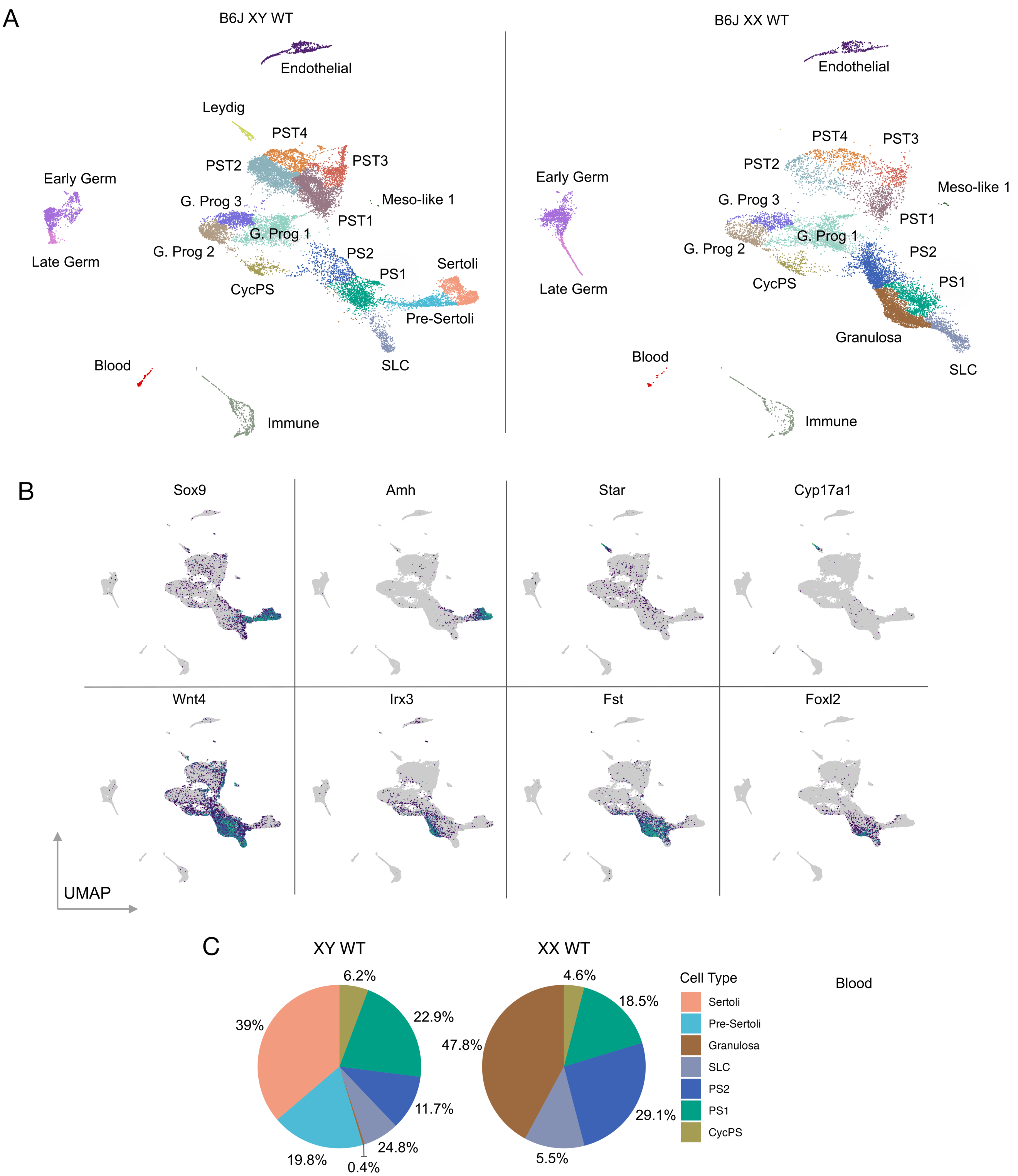

Figure S1

Development • Supplementary information

Fig. S1. UMAP presentations by stage, gene expression and proportions. A) UMAPs of B6J XY (left) and XX (right) wild-type (WT) cells, showing sexual dimorphism. B) Expression of eight marker genes of supporting and steroidogenic cell lineages shown on all-cell UMAP. C) Pie-charts showing proportions of distinct cell- types in XX and XY WT gonads. Blue cells, lower gene expression; green cells, higher gene expression. G. Prog, gonad progenitor; PS1, pre-supporting 1; PS2, pre- supporting 2; SLC, supporting-like cells; CycPS, cycling pre-supporting.

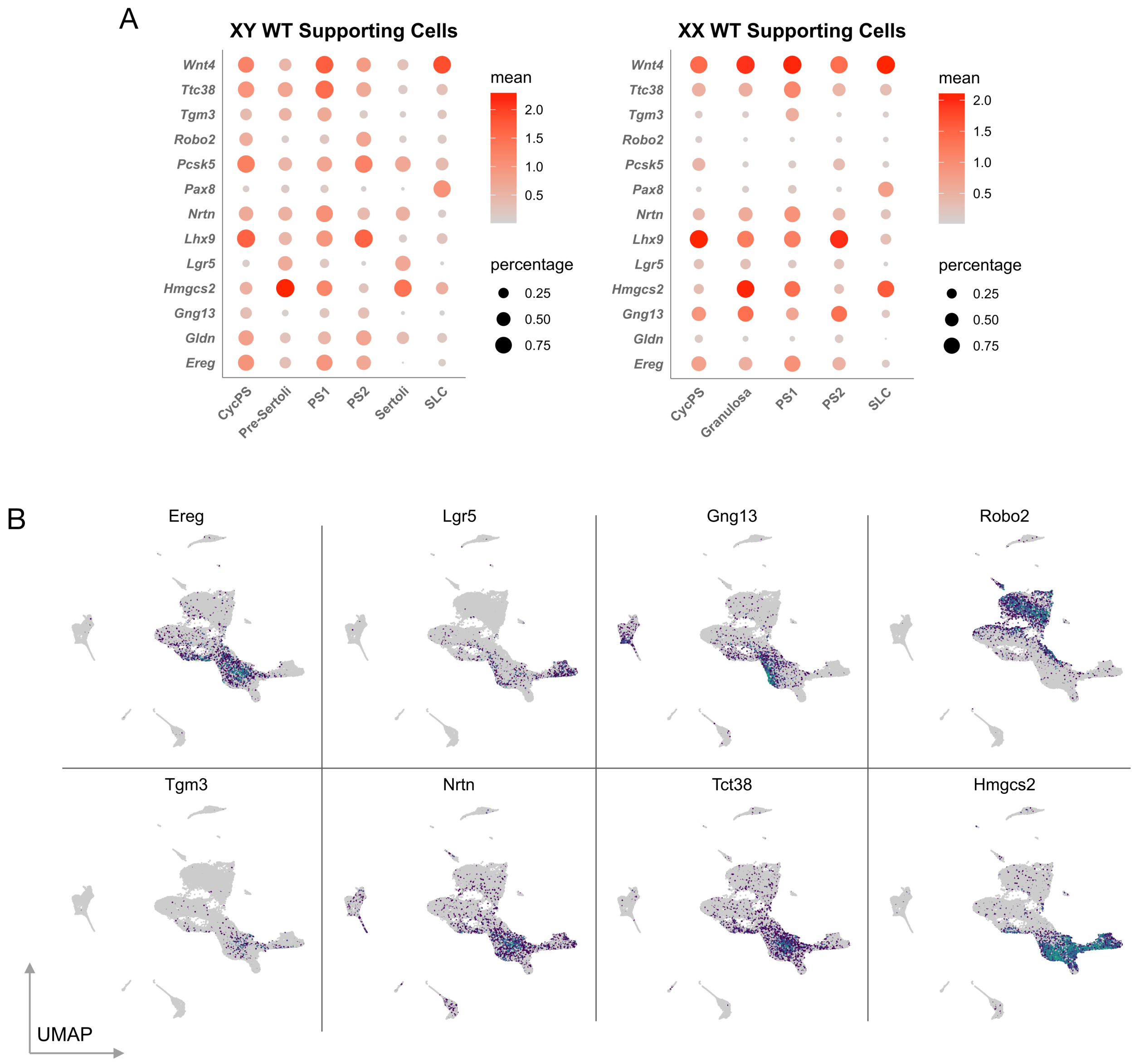

Figure S2

Fig. S2. Expression of pre-supporting (PS) lineage marker genes in supporting cell-types. A) Dot-plot of B6J XX and XY WT gene expression, at three stages (11.5, 12.5 and 14.5 dpc) combined. B) All-cell UMAP showing expression of eight PS lineage markers genes. Many cell markers distinguish PS1 from PS2 in XX WT, including *Gng13* and *Hmgcs2*. *Robo2* and *Pcsk5* distinguish PS1 from PS2 in XY WT. Blue cells, lower gene expression; green cells, higher gene expression.

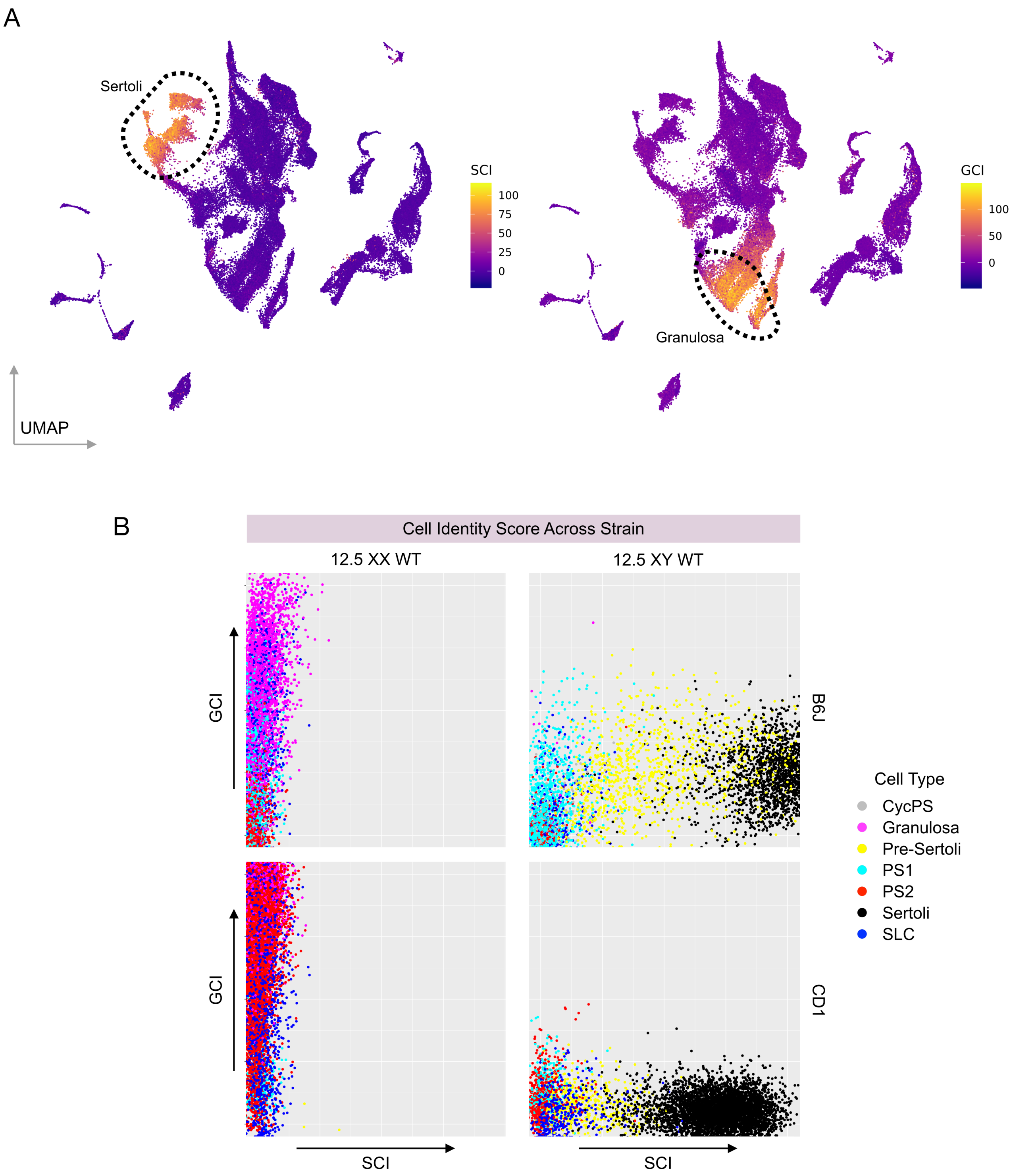

Figure S3

Fig. S3. Combined B6J and CD1 datasets (at 12.5 dpc) and supporting cell sex identity in these two strains. A) Combined CD1 and B6J UMAPs with Sertoli cell identity (left, SCI) and granulosa cell identity (right, GCI) scores indicated by colour (key on far right). B) Plots of GCI (y-axis) and SCI (x-axis) scores in XX (left) and XY (right) WT supporting cells in B6J (upper) and CD1 (lower), with cell identity indicated by colour (key on right).

Development • Supplementary information

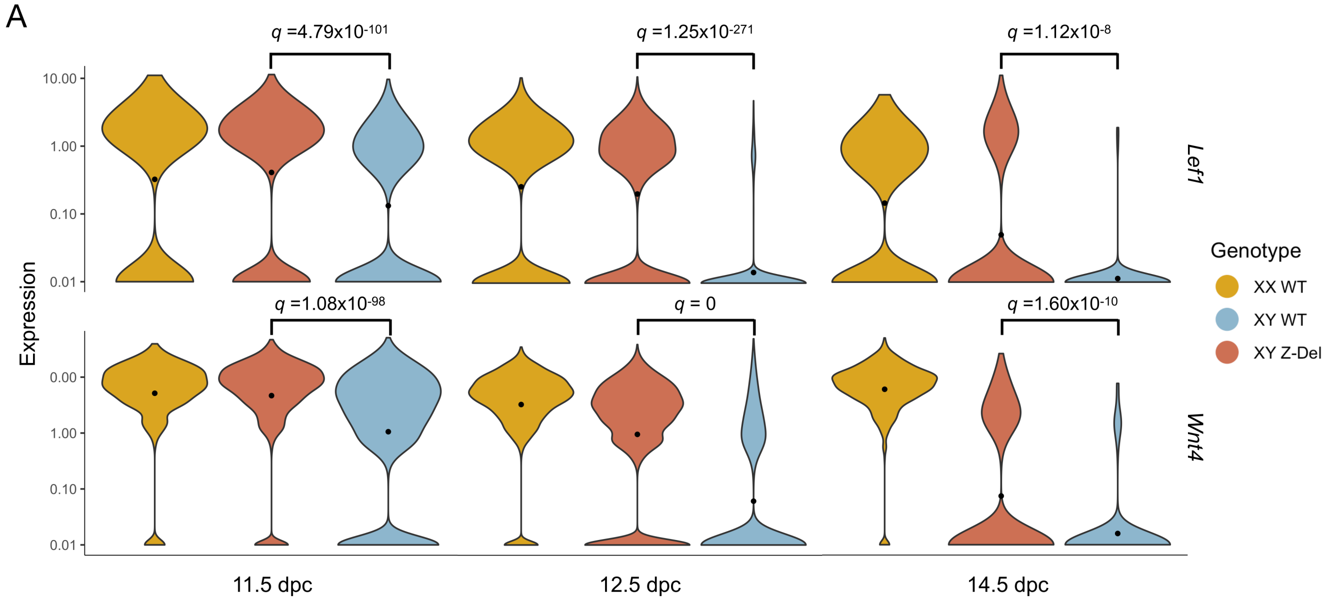

Figure S4

Fig. S4. *Wnt* gene expression in supporting cells of B6J XY wild-type (WT), XX WT and XY *Z*-del gonads at three stages (11.5, 12.5 and 14.5 dpc). Significant differential expression was seen between XY WT and XY Z-del at each stage in both Wnt4 and Lef1.

Development • Supplementary information

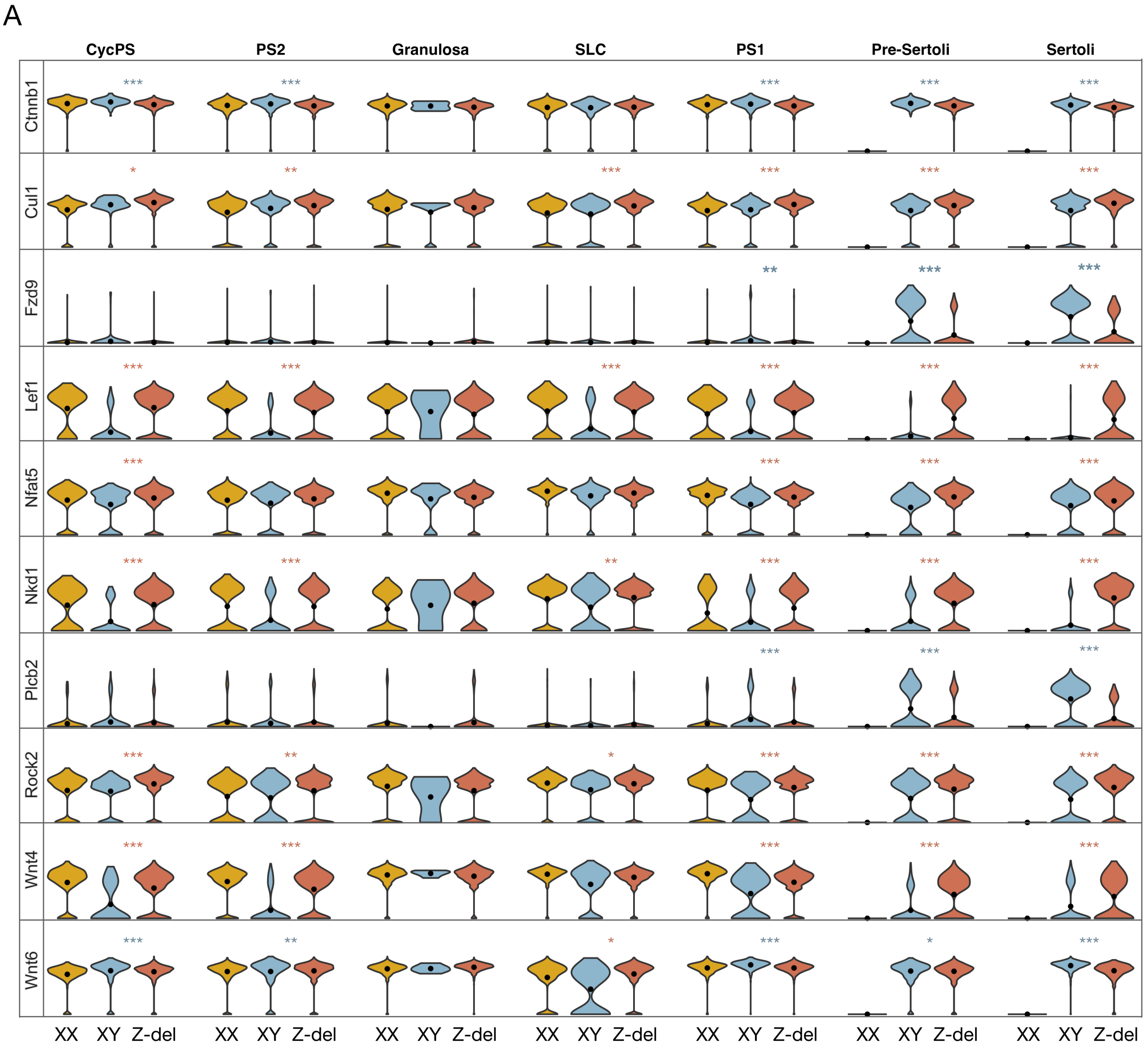

Figure S5

Fig. S5. Top 10 differentially expressed WNT pathway genes in the supporting cells between, B6J XY WT and XY Z-del at 12.5dpc. XY WT (blue), XY Z-del (red) and XX WT expression (yellow). Significantly dysregulated WNT genes are denoted by stars: 1 star, q=0.05-0.01, 2 stars, q=0.01-0.001, and 3 stars q<0.001, with colours indicating the direction of regulation: blue, expression is significantly lower in Z-del compared to XY WT, and red, expression is significantly higher in XY Z-del compared to XY WT. Fzd9 and Plcb2, show XY lineage specific differences.

Development • Supplementary information

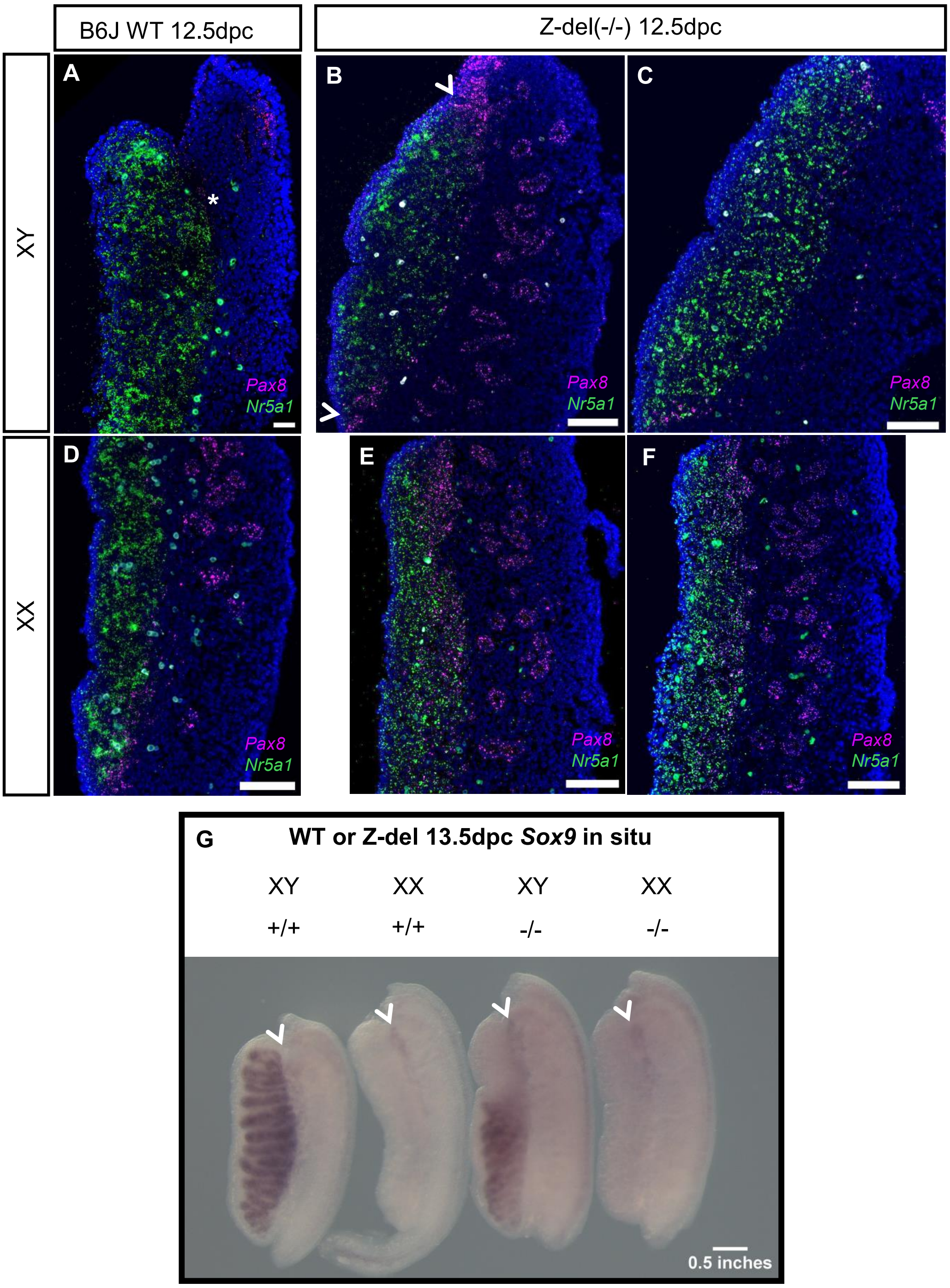

Figure S6

Development • Supplementary information

Fig. S6. *Pax8* expression is elevated in mutant ovarian tissue but no overt rete abnormalities are observed. A) RNAscope with *Pax8* (magenta) and *Nr5a1* (*Sf1*, green, gonadal somatic cell marker) in section of B6J XY WT gonad at 12.5 dpc reveals weak *Pax8* expression in anterior region of gonad, particularly in the area of the developing rete testis (asterisk). Panels B) and C) show elevated *Pax8* expression at the poles (arrowheads in B) of two XY *Z*-del gonads, also at 12.5 dpc. D) *Pax8* expression in XX WT gonadal tissue is comparable to XY WT. In XX *Z*-del (E, F), *Pax8* expression is more prominent, particularly in areas adjacent to the mesonephros (gonad: left, mesonephros: right), which lacks *Nr5a1* expression. *Pax8* expression can also be observed in mesonephric tubules. G) Wholemount *in situ* hybridisation of 13.5dpc XY WT (+/+), XX WT (+/+), XY *Z*-del (-/-), and XX Z-del (-/-) gonads with a *Sox9* probe. *Sox9* is a marker of the testicular Sertoli cells in XY WT gonads, but can also be seen, at lower levels, in the region of the rete testis and ovarii in all samples (arrowheads). Scale Bar in panels A-F equal 50μm.

Development • Supplementary information

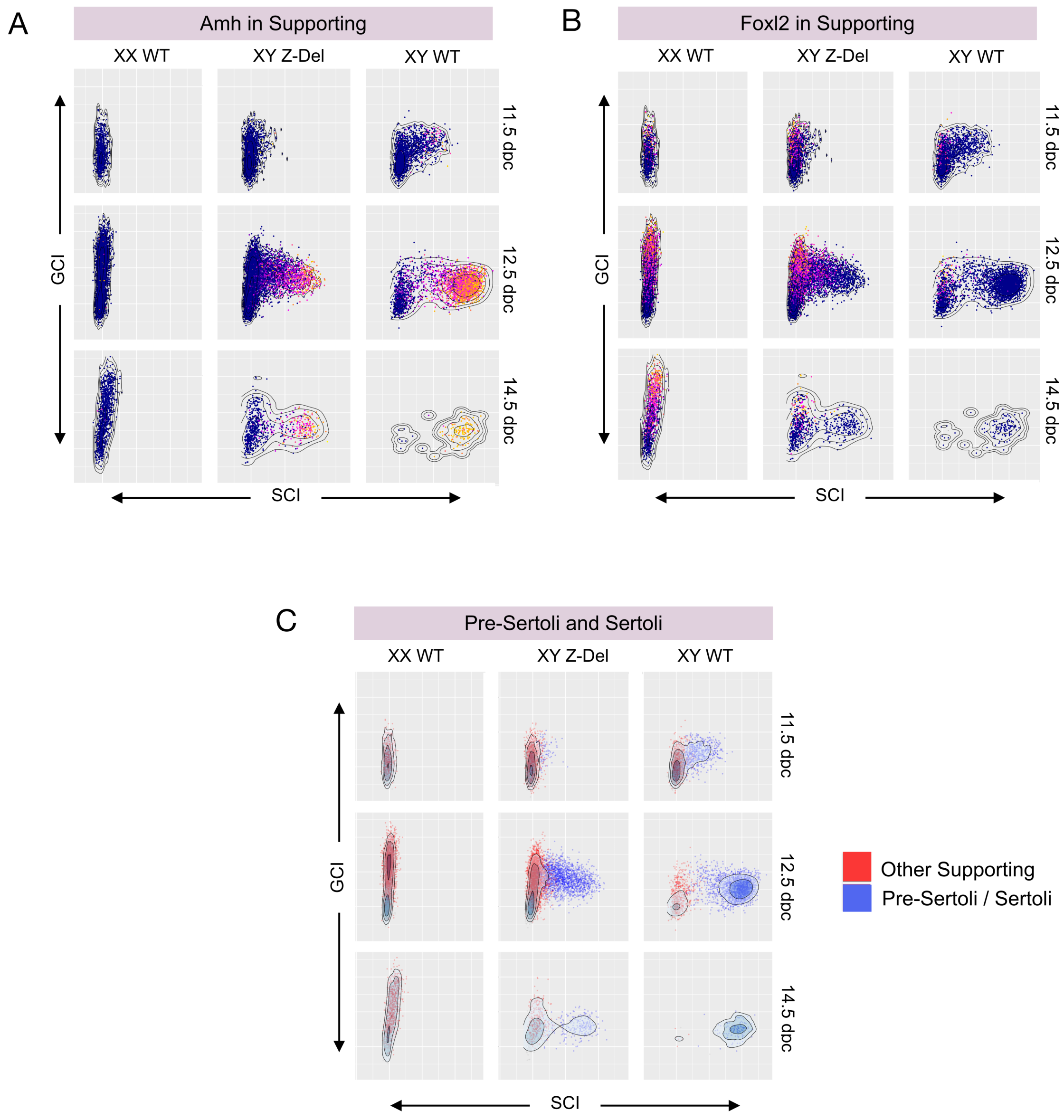

Figure S7

Fig. S7. Expression of Sertoli (*Amh*) and granulosa marker (*Foxl2*) in supporting cells plotted against GCI and SCI scores. A) *Amh* expression is prominent at 12.5 and 14.5 dpc in B6J XY wild-type (WT) cells with high SCI scores (Sertoli cells). Many fewer cells achieve *Amh*-positive Sertoli cell status in XY *Z*-del gonads. B) *Foxl2* expression marks cells with high GCI and low SCI scores in XX WT gonads at all stages. Large numbers of *Foxl2*-positive cells are still observed at 14.5 dpc in XY *Z*-del gonads, in contrast to XY WT. C) The identity of supporting cells on the GCI-SCI plot indicated by marking of cells derived from the pre-Sertoli/ Sertoli clusters (blue) and all other supporting cell types (red).

Development • Supplementary information

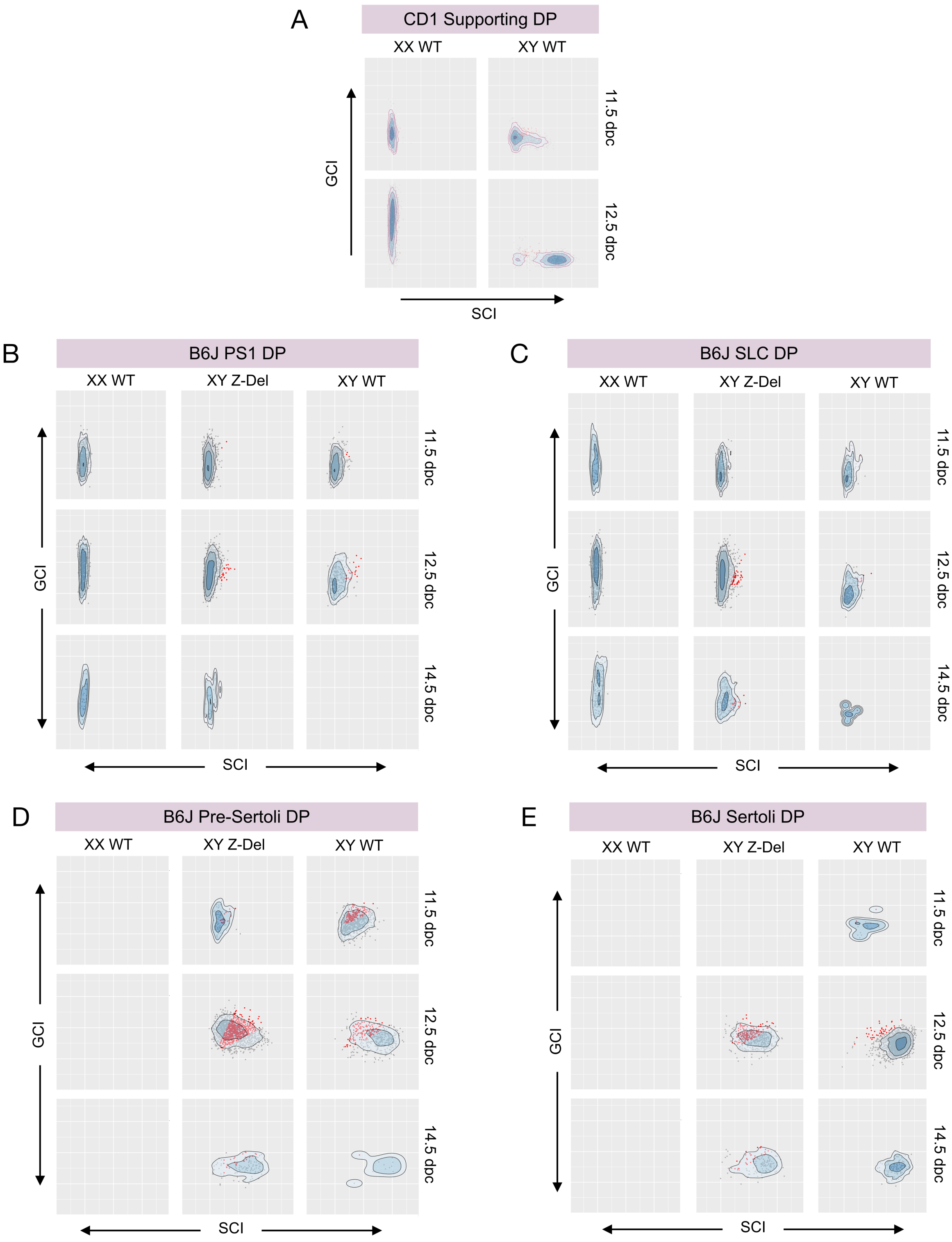

Figure S8

Fig. S8. Double-positive (DP) cells (red) in distinct gonadal supporting cell lineages shown on GCI/SCI scatter-plots. A) DP cells in CD1 supporting cells at 11.5 and 12.5 dpc. DP cells in all B6 genotypes at all stages in B) Pre-supporting cluster 1 (PS1) cells, C) Supporting-like cells (SLC), D) Pre-Sertoli cells, and E) Sertoli cells.

Development • Supplementary information

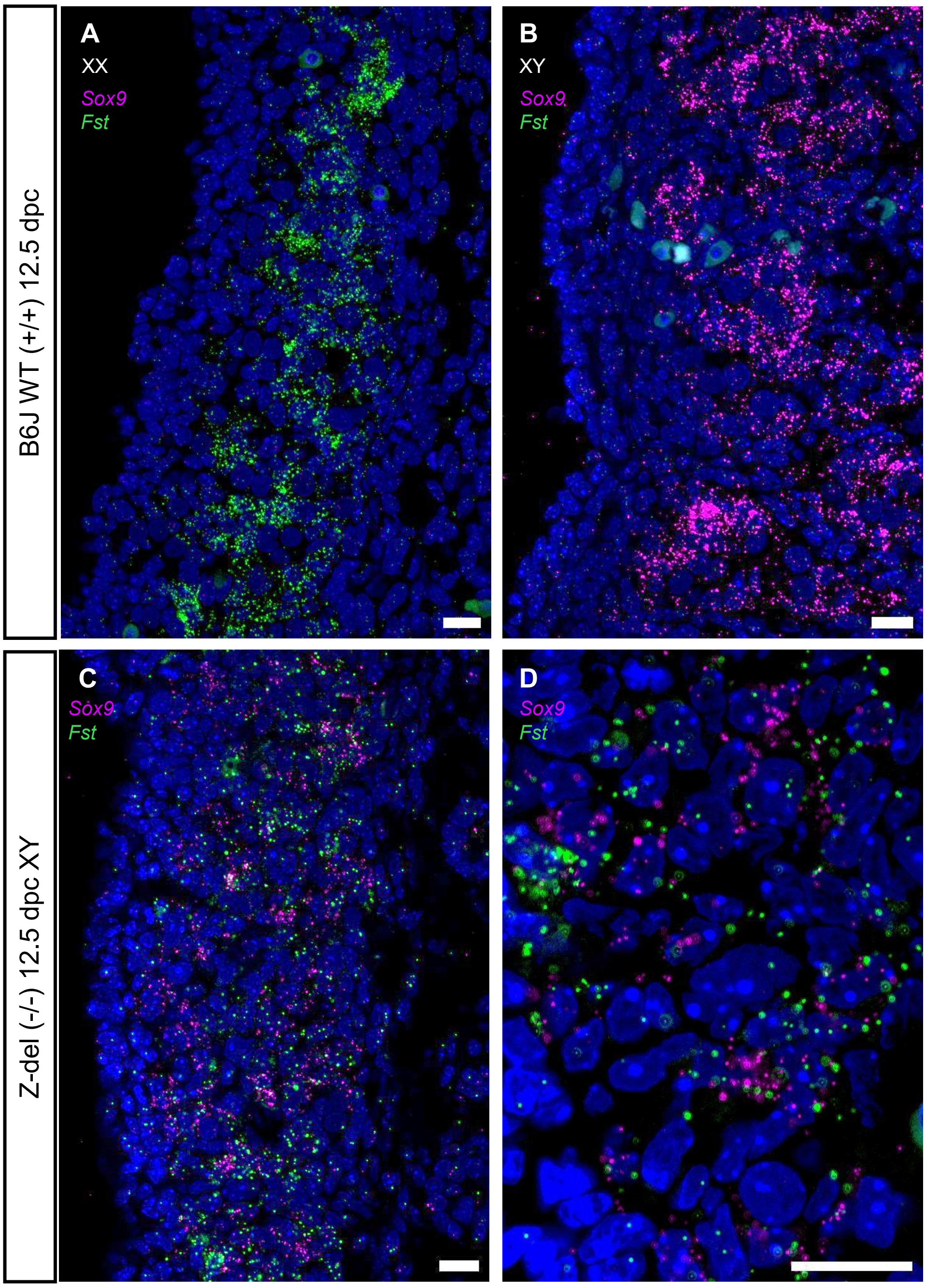

Fig. S9. Visualisation of *Sox9* and *Fst* in B6J wild-type (+/+) and XY *Z*-del (-/-) gonads at 12.5 dpc. RNAscope *in situ* hybridisation of *Sox9* (magenta) and *Fst* (green) of A) longitudinal XX WT gonad section and B) XY WT gonad section, showing sex-specificity of each gene. C) Hybridisation of same gene probes to gonad sections from XY *Z*-del mutants, showing expression of both genes, where D) is at higher magnification, showing cellular co-expression. Scale bars in all panels are 20µm.

Development • Supplementary information

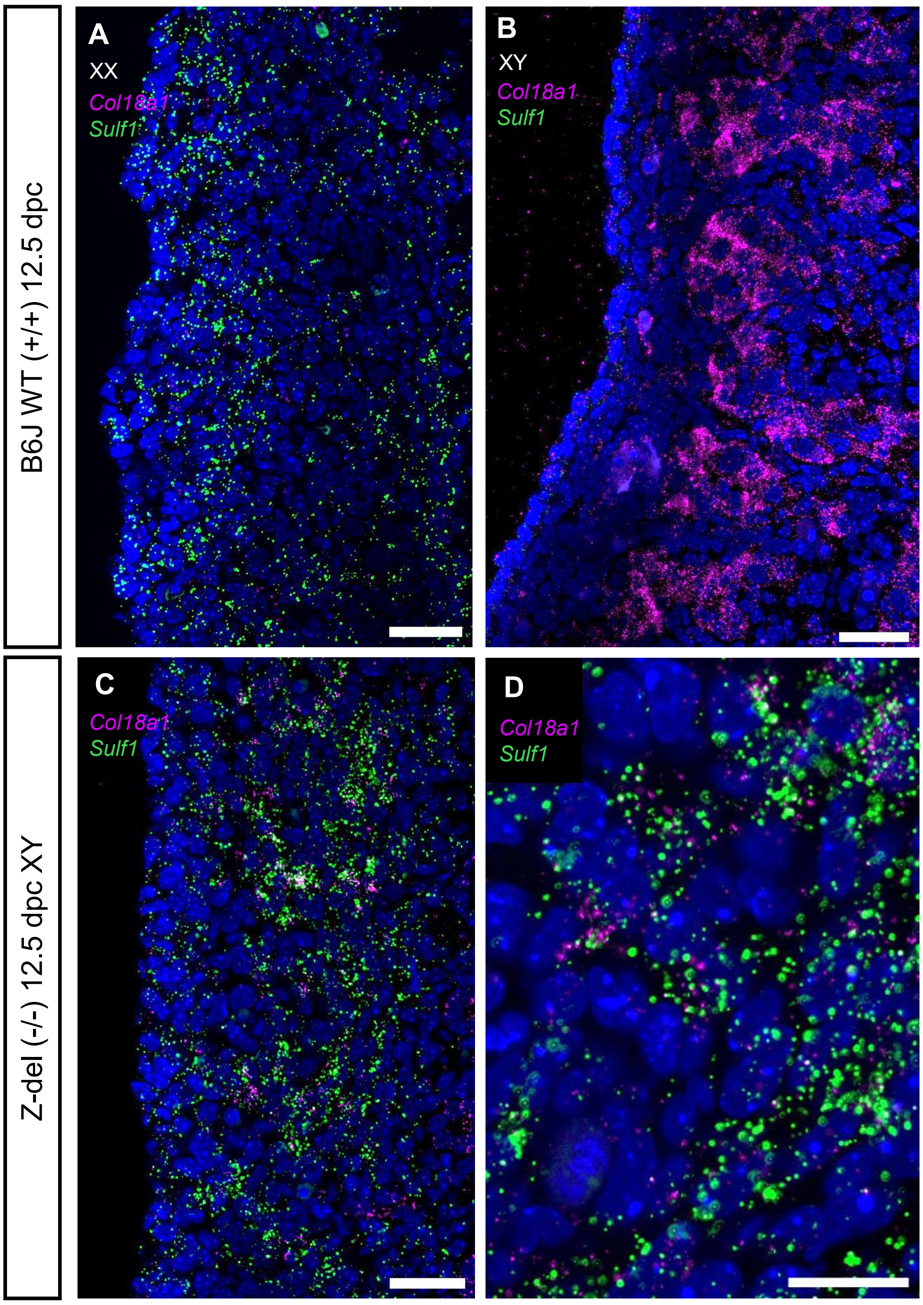

Fig. S10. Visualisation of *Col18a1* and *Sulf1* in B6J XY WT (+/+) and XY *Z*-del (-/-) gonads at 12.5 dpc. RNAscope *in situ* hybridisation of *Col18a1* (magenta) and *Sulf1* (green) of A) longitudinal XX WT gonad section and B) XY WT gonad section, showing sex-specificity of each gene. C) Hybridisation of same gene probes to gonad sections from XY *Z*-del mutants, showing expression of both genes, where D) is at higher magnification, showing cellular co-expression. Scale bars in all panels are 20μm.

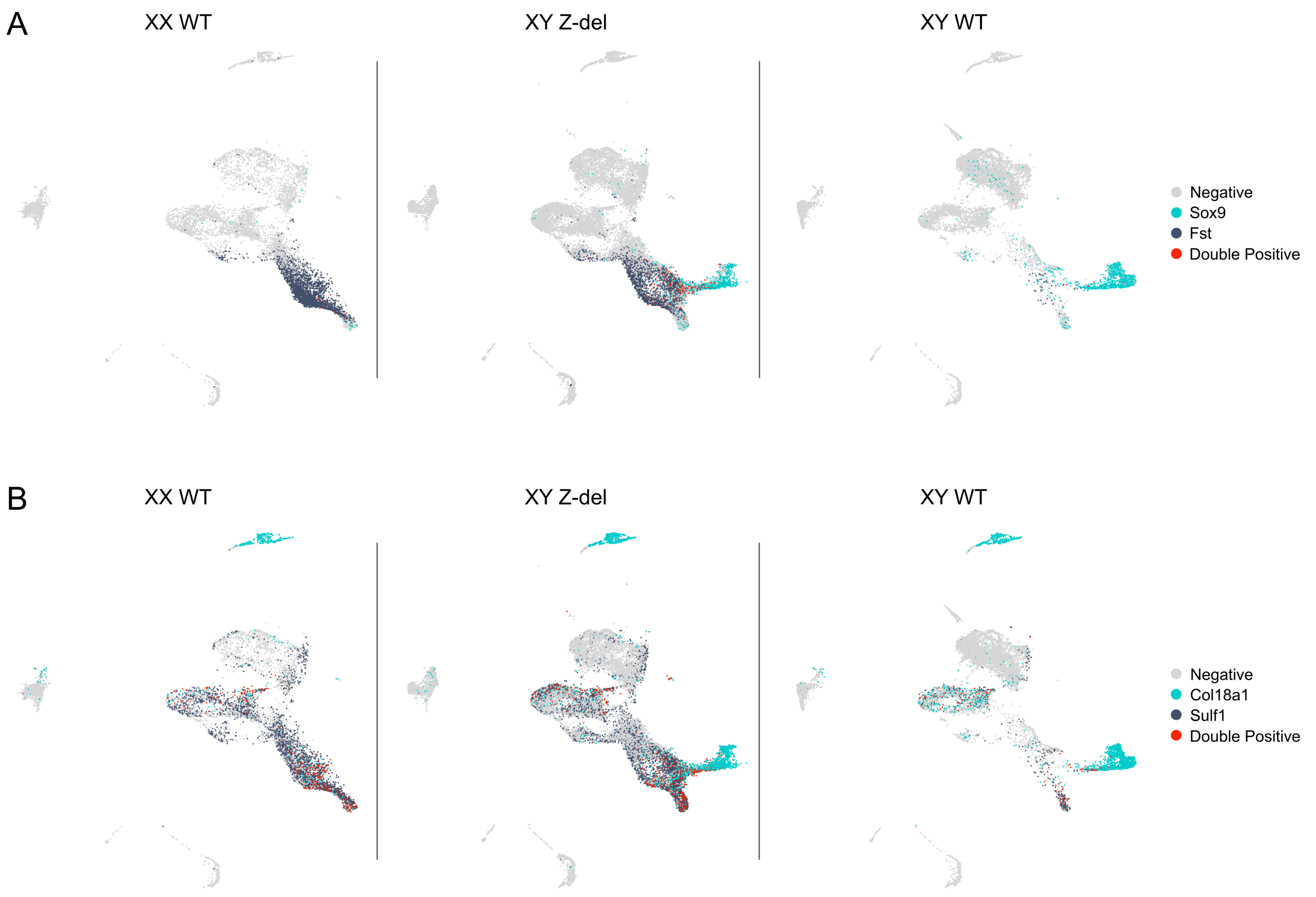

Figure S11

Fig. S11. UMAPs showing location of B6J double-positive (DP) cells. A) Expression of *Sox9* and *Fst* on all-cell UMAP, with cells expressing both genes (double positive) indicated in red. B) Expression of *Col18a1* and *Sulf1* on all-cell UMAP, with cells expressing both genes (double positive) indicated in red.

Development • Supplementary information

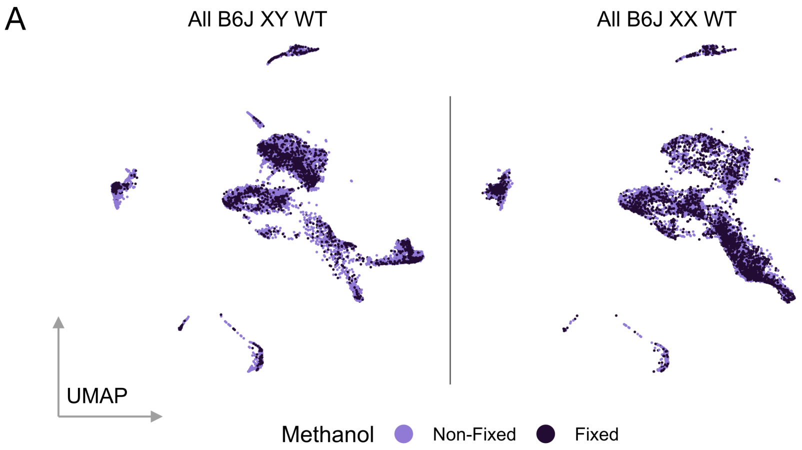

Figure S12

Fig. S12. UMAP of methanol-fixed and non-fixed B6J gonadal cells. The all- cell UMAP shows that methanol fixation does not adversely affect the quality of single- cell transcriptomic analyses.

Development • Supplementary information

Table S1. Proportions of distinct cell-types in XY *Z*-del mutant and B6J control (XX and XY wild-type (WT)) gonads, per sample, at all stages.

Available for download at
https://journals.biologists.com/dev/article-lookup/doi/10.1242/dev.202707#supplementary-data

Table S2. Gene expression signatures defining Sertoli cell and granulosa cell differentiation in B6J and CD1 WT gonads.

Available for download at
https://journals.biologists.com/dev/article-lookup/doi/10.1242/dev.202707#supplementary-data

Table S3. Number of cells per cycle phase at each time point in each supporting cell type cluster in B6J XY Z-del, B6J XY WT, and B6J XX WT.

Available for download at
https://journals.biologists.com/dev/article-lookup/doi/10.1242/dev.202707#supplementary-data

Table S4. Differential gene expression of classical Wnt genes between B6J XY Z-del and B6J XY WT.

Available for download at
https://journals.biologists.com/dev/article-lookup/doi/10.1242/dev.202707#supplementary-data

Table S5. Comparison of proportions of distinct cell-types in XY Z-del mutant and B6J XY wild-type gonads, per genotype, at all stages.

Up_In	Cell_Type	Stage	XY_WT_perc	XY_Z-del_perc	pvalue	qvalue
XY_WT	Granulosa	11.5 dpc	0.722	0.605	0.720092616	0.720092616
XY_WT	Pre-Sertoli	11.5 dpc	26.9	2.48	3.74E-161	3.93E-160
XY_WT	Sertoli	11.5 dpc	0.812	0	7.46E-07	1.31E-06
XY_WT	Sertoli	12.5 dpc	58.4	4.57	0	0
XY_WT	Sertoli	14.5 dpc	84.7	18.6	1.90E-51	7.97E-51
XY_Z-del	CycPS	11.5 dpc	9.16	11.8	0.002467222	0.003700832
XY_Z-del	PS1	11.5 dpc	40	58.5	6.01E-41	1.80E-40
XY_Z-del	PS2	11.5 dpc	14.7	16.8	0.035055426	0.046010247
XY_Z-del	SLC	11.5 dpc	7.72	9.86	0.007263605	0.010169047
XY_Z-del	CycPS	12.5 dpc	3.63	10.1	1.04E-30	2.44E-30
XY_Z-del	Granulosa	12.5 dpc	0.183	6	2.17E-43	7.58E-43
XY_Z-del	Pre-Sertoli	12.5 dpc	13	13.3	0.709051975	0.720092616
XY_Z-del	PS1	12.5 dpc	9.45	18.5	4.76E-34	1.25E-33
XY_Z-del	PS2	12.5 dpc	8.66	25.8	3.53E-95	2.47E-94
XY_Z-del	SLC	12.5 dpc	6.74	21.8	4.07E-84	2.14E-83
XY_Z-del	CycPS	14.5 dpc	0	3.3	0.078195225	0.086426301
XY_Z-del	Granulosa	14.5 dpc	0	8.06	0.002030879	0.00328065
XY_Z-del	Pre-Sertoli	14.5 dpc	8.87	16	0.05466768	0.066951994
XY_Z-del	PS1	14.5 dpc	0	3.7	0.057387423	0.066951994
XY_Z-del	PS2	14.5 dpc	1.61	23.1	5.99E-08	1.26E-07
XY_Z-del	SLC	14.5 dpc	4.84	27.2	1.23E-07	2.34E-07

Table S6. Proportions of double-positive cells in XY *Z*-del mutant and B6J control (XX and XY wild-type (WT)) gonads at all stages in distinct cell-types.

Available for download at
https://journals.biologists.com/dev/article-lookup/doi/10.1242/dev.202707#supplementary-data

Table S7. Average SCI/GCI scores and significance in supporting cell-types in XY Z- del mutant and B6J XY wild-type, at 12.5 dpc and 14.5 dpc.

Available for download at
https://journals.biologists.com/dev/article-lookup/doi/10.1242/dev.202707#supplementary-data

Table S8. Differential expression of three gonadal cell-associated genes: *Foxl2, Pax8 and Amh, at all stages in XY Z-del mutant and B6J XY wildtype gonads.*

	Stage	Cell Type	significance	fold-change
Foxl2:	E11.5	SLC	8.15E-06	1.205842222
	E12.5	SLC	9.00E-18	1.386726266
	E14.5	SLC	0.986035358	15.22173299
	E12.5	Sertoli	2.21E-20	3.244295139
	E14.5	Sertoli	0.991030068	17.72112142
Amh:	E11.5	SLC	0.535567514	-0.357551752
	E12.5	SLC	1.06E-12	-1.530117983
	E14.5	SLC	0.993454005	15.27054576
	E12.5	Sertoli	5.23E-85	-1.66912629
	E14.5	Sertoli	2.54E-39	-1.64783668
Pax8:	E11.5	SLC	0.110485503	-0.146945482
	E12.5	SLC	2.81E-37	-0.797777251
	E14.5	SLC	0.017680071	-1.206624154
	E12.5	Sertoli	1.53E-05	1.787470613
	E14.5	Sertoli	1	-9.83E-13

Development • Supplementary information
