# Supplementary material for: Gonadal sex reversal at single-cell resolution in Znrf3-deficient mice
Source: Development. 2024 Dec 4;151(23):dev202707. doi: 10.1242/dev.202707 (PMC11658683; doi:10.1242/dev.202707)
Supplement: Supplementary file 1 [file develop-151-202707_review_history.pdf]

Gonadal sex reversal at single-cell resolution in *Znrf3*-deficient mice

Raissa GG Kay, Richard Reeves, Pam Siggers, Simon Greenaway, Ann-Marie Mallon, Sara Wells, Bon-Kyoung Koo, Chloe Mayere, Serge Nef, Andy Greenfield, and Michelle M. Simon
DOI: 10.1242/dev.202707

Editor: Anna-Katerina Hadjantonakis

Review timeline
Original submission: 8 February 2024
Editorial decision: 25 March 2024
First revision received: 6 August 2024
Accepted: 6 September 2024

Original submission

First decision letter

MS ID#: DEVELOP/2024/202707

MS TITLE: Gonadal sex reversal at single-cell resolution in Znrf3-deficient mice

AUTHORS: Raissa GG Kay, Richard Reeves, Pam Siggers, Simon Greenaway, Ann-Marie Mallon, Sara Wells, Bon-Kyoung Koo, Chloe Mayere, Serge Nef, Andy Greenfield, and Michelle M Simon

I have now received all the referees' reports on the above manuscript, and have reached a decision. The referees' comments are appended below, or you can access them online: please go to BenchPress and click on the 'Manuscripts with Decisions' queue in the Author Area.

As you will see, the referees express considerable interest in your work, but have some significant criticisms and recommend a substantial revision of your manuscript before we can consider publication. If you are able to revise the manuscript along the lines suggested, which may involve further experiments, I will be happy receive a revised version of the manuscript. Your revised paper will be re-reviewed by one or more of the original referees, and acceptance of your manuscript will depend on your addressing satisfactorily the reviewers' major concerns. Please also note that Development will normally permit only one round of major revision. If it would be helpful, you are welcome to contact us to discuss your revision in greater detail.

Reviewer 1

Advance summary and potential significance to field

The process of sex determination relies on a delicate balance between two antagonistic gene regulatory networks of pro-male versus pro-female factors. While we know that disturbance in this delicate balance or to any of the key factors would cause sex reversal in mice and DSD in human, we do not really understand well, at the molecular level, the events that take place.
In this beautiful paper, kay et al., provide a detailed transcriptomic analysis of a strain of mice carrying mutation in the ZNRF3 factor, leading to inability to form normal males. It is nice that they chose to thoroughly analyse these mice as there is variability in severity of phenotypes among

© 2024. Published by The Company of Biologists under the terms of the Creative Commons Attribution License (https://creativecommons.org/licenses/by/4.0/).

different animals and even within the same animal, ranging from XY females, XY ovotestis and XY males.
In this study, the authors performed scRNA-seq analysis of WT XY, XX and XY ZNRF3 mutants at 3 relevant developmental timepoints: E11.5, E12.5 and E14.5. They show that mutant XY gonads are less able to differentiate to the Sertoli lineage, remaining more at the precursor stage.
Furthermore, many cells are co- expressing male and female factors as evident by the RNA-seq and RNA-scope. They also see that more cells in the mutants differentiate into the Pax8+ SLC lineage instead.
ZNRF3 has a role at degrading the WNT pathway and in its absence, there is more Wnt4 expression in XY gonad, leading to the sex reversal.
In addition to showing transcriptomic description of mice with variable sex reversal phenotypes, they also provide the first scRNA-seq data done on C57BL/6J background. The existing scRNA-seq data was done on a CD1 background.
This is important data as many mice strains are being generated on C57BL/6J background and in addition the C57BL/6J mice are known to be more feminized than other lines. While the mechanism behind was shown before by other groups, there was no scRNA-seq transcriptomic data on this.
The authors performed an in depth analysis comparing the CD1 and C57BL/6J transcriptomic and show that the C57 mice have higher levels of pro-female genes like Wnt4 and Lef1, among others, explaining this phenomenon. I believe that this transcriptomic data will be useful for many researchers in the field.

The manuscript is very nicely written, clean and concise and very fluent to read.

Comments for the author

I have a few minor comments:

1. When describing in the introduction the pro-female factors it may be nice to also include RUNX1 (Yao's lab) and the more recently characterized WT1 (Chaboissier lab).

2. In figures S8 and S9 - please improve the labelling of the figures: please add scale bars and mention in the figure the stage of the gonads. The D in both (bigger magnification)- is missing the labelling of the RNA-scope markers.

3. In figure S5- again- scale bars and stages but also in G- the labelling is not clear- It is not clear that these are ZNRF3 KO and it is a Sox9 in situ. The figure should be self-explanatory even without the figure legend.

<u>Reviewer 2</u>

Advance summary and potential significance to field

In this paper the authors analysed Znrf3 mutant mouse gonads at single cell resolution. This work provided interesting insight into the molecular perturbations behind the Znrf3 phenotype and the observed phenotypic variability. Not surprisingly, the authors found that this included significant disruption to male somatic cell differentiation in particular from supporting lineages to Sertoli lineages, but also with evidence of accumulation to a recently described SLC population. Of interest was the observation that a number of cells also expressed markers of both female and male somatic fate (Granulosa and Sertoli), however this was not confirmed at protein level so the functional consequences of this remain unclear. Overall, this work provides a significant and novel understanding of sex development of the gonads and how it can be disrupted, which is of interest to all researchers in the sex differentiation field including those working in human reproductive conditions. It also revealed why a B6J genetic background is sensitised to sex development aberrations. Specifically, it suggested that cell lineages and gene expression are somewhat feminized in this background compared to other less sensitive strains. This will be of broad interest to reproductive biologists and those who use murine models of sex development.

Comments for the author

© 2024. Published by The Company of Biologists under the terms of the Creative Commons Attribution License (https://creativecommons.org/licenses/by/4.0/).

I found the work compelling and well presented, the conclusions were well justified and the paper beautifully written. I have only minor suggestions and amendments.

1. It was interesting to see the differences in Wnt and Lef expression in the different genetic backgrounds. I found myself wondering about the single cell expression of Znrf3 and other pathway members in both the WT/mutant groups of each genetic background. Some of these are shown for example in Figure 1, but could expression across cell types (UMAP or dot plot) be provided for Znrf3, Rnf43, Fzd and Wnts as well as downstream effectors in both genetic backgrounds (WT and mutant). Are there differences in expression of these that are cell lineage- specific?

2. The differences observed of Sertoli cells in the G1 cell cycle phase in the Z-del gonads compared to WT made me wonder about the total number of resulting Sertoli + Granulosa (or in fact total number of any somatic cells) in these gonads. I could not see this info provided in any of the tables – but it would be worth commenting on. Is there an overall change in cell numbers? Or just Sertoli proliferation? Are cells committing but not proliferating? Is this exacerbated over time?

3. I understand the focus here is on somatic gonadal cells, however I did find it interesting that in Figure 2B – Germ cell 2 cluster missing in B6J XY c.f CD1 XY. It isn't totally clear to me what the different Germ cell clusters (1,2,3) are here, but this may also be evidence that in addition to B6J showing "feminization" that there may also be delayed maturation (does Germ cell 2 represent those undergoing mitotic arrest?). Similarly, Germ cell pop 3 is missing at E12.5 in XX B6J. Why?

Minor queries and amendments:

1. Although it may be explicit in the text, each Figure and Figure legend should note the genetic background represented. E.g. Figure 1 – BL6.

2. Page 6 results/methods – is n gonads or gonad pairs?

3. Figure 2A. The different cellular lineages are clear and represented in Figure 1 however in Figure 2A it isn't clear what Germ 1, 2, and 3 refer to and what the gene expression profiles defining these are.

4. Need to be consistent with case used for Figures and Figure legends (A vs. a)

5.Figure 2 C. Consider expression of other WNT pathway markers too including Znrf3?

6.Figure 2 D. I found this figure difficult to interpret. Consider labelling the four subplots/quadrants ?

7.Figure 4 A. The figure legend says XY WT but in Figure it says XY Z-Del 12.5 dpc S12. In fact, this heading is used for all velocity plots in this Figure – assuming this is an error, need to revise to indicate the presence of different genotypes and or different samples.

8.Figure S6C. This plot appears to be exactly the same as S6B? Just Amh again? Revise to show Foxl2?

<u>Reviewer 3</u>

Advance summary and potential significance to field

This paper provides single cell transcriptomic analysis of partial sex reversal in Znfr3 mutant mice. Insight is provided into the initiation of sex determination.

Comments for the author

In this paper, Kai and Reeves et al examine the transcriptional changes associated with sex reversal in XY Znrf3 mutant mice of C57B6L/6J (B6J) background.

© 2024. Published by The Company of Biologists under the terms of the Creative Commons Attribution License (https://creativecommons.org/licenses/by/4.0/).

Previously, the group identified that ZNRF3 is required for testis determination, with deletion resulting in reduced Sox9 expression at E11.5 and gonadal sex reversal. This current submission is a further interrogation of Znrf3 involvement in supporting and supporting-like cell (SLC) fate in the fetal gonads. Single-cell RNAseq is used to investigate the transcriptional profile of gonads during and after sex differentiation (E11.5, E12.5, E14.5) in wild- type XX, XY, and Znrf3 mutant mice.

The authors confirm several previously published observations, for example that Sox9 is expressed both in pre-Sertoli and Sertoli cells, that Foxl2, Fst and Irx3 are detected in granulosa cells, and that the ovary markers Wnt4 and Lef1 are more highly expressed in B6J XY than CD1 gonads. There are some interesting novel findings, e.g. that the testis marker Col18a1 and ovary marker Sulf1 are co-expressed in XY Znrf3 mutant gonads, and the high heterogeneity of supporting cells and SLCs in Znrf3 mutant gonads, highlighting the variability of cellular sex identity in this mutant. The authors also used machine learning to score and assess similarity of cells to Sertoli or granulosa cell identity. They compare their findings in wild-type B6J XX and XY mice to previously published data on the CD1 mouse strain and find that there is a higher number of pre-supporting cells in the XY gonads of B6J than of CD1 strain. However, since the role of supporting cells in sex determination is well-established, and the authors have previously published on the sex- reversal of Znrf3 mutants, the novelty and implications of their current findings are not thoroughly described, and the manuscript would benefit from further analyses or clarification to enhance its significance. Comments and suggestions are listed below.

Major comments:

1. Currently, the study's scope is narrow and highly descriptive, the analysis lacks clear relevance and context. There are issues with grammar/sentence structure and following the narrative is challenging, and the nomenclature is inconsistent (e.g. supporting cell precursor vs pre-Sertoli and pre- granulosa). There is potential to expand the discussion on other candidate genes affected by Znrf3 KO.

2. There are concerns related to reproducibility, including the Github link provided by the authors, which is currently empty, and missing supplementary bioinformatic methods. Furthermore, the authors describe using a dataset containing 68,000 cells. This dataset contained three genotypes (XX, XY, XY Znrf3 KO), at three timepoints (11.5, 12.5 and 14.5), however there's no breakdown of how many cells there were per genotype / timepoint. Are all three genotypes and timepoints represented equally in terms of cell number? They give a breakdown of proportions per cluster in Fig 2B, however it's harder to interpret if we don't know what the timepoint proportions are. Clarification is required on the filtering of LQ cells. They state that Seurat was used to remove low quality cells based on genes expressed, counts etc. Under "Filtering of Low-Quality Cells" they describe that thresholds were changed per sample due to sequencing depth, but then give hard thresholds, i.e. "Cells with UMI counts of <2% and >98% were removed", so were these thresholds applied to all cells, regardless of their sequencing depth? Following on, in the section "Cell clustering and cell-type annotation", they then apply more filtering by removing "Cells within the bottom 5% percentile of counts (~5000 counts)", with the aim of removing small UMAP clusters. But doesn't this mean two rounds of LQ filtering have occurred? Usually, the initial LQ filtering should be enough as they already excluded low counts cells, but then they do it again to make the UMAP?

3. A clarification is required on the background that B6J strain was compared to in this study. Mayere et al study used mixed 129/CD1 strain rather than pure CD1 background. Furthermore, Mayere et al, 2021 bioRxiv reference is missing from bibliography.

4. The figures require major attention, for example:

Fig1C: The large dotplot showing marker expression per UMAP cluster does re-enforce that the clusters have indeed been annotated correctly, however this kind of figure would be better suited in supplementary. Page 40, figure 1 C – it would be easier to interpret this dot plot if it would highlight appropriate sections for specific populations or marker genes.

Page 40, figure 1 B – this would benefit from separate UMAPs of different time points. It is unclear if some cells from different timepoints overlap.

© 2024. Published by The Company of Biologists under the terms of the Creative Commons Attribution License (https://creativecommons.org/licenses/by/4.0/).

Fig2B: The barplots showing proportions per cluster. There are 18 clusters, which makes interpretation of this figure difficult. Again, would be better suited in supplementary, or simplified by combining numbered clusters, i.e. “Germ1”, “Germ2”, “Germ3” = “Germ”

Fig2D: It’s not clear what each individual scatter plot represents. For example, the bottom right scatter plot is more SCI than GCI, and the bottom left is low in both SCI and GCI, but what is the relationship between each plot? How are these discrete plot boundaries decided? Page 41, figure 2 D – it is not clear how the scatter plot is divided into quarters and what the significance is.

Page 10, “A principal component analysis of these proportions (Fig.3d, Table S3) shows that XY Z-del mutants at 12.5 dpc group with 11.5 dpc XX and XY wild-type samples, suggesting a failure of commitment to testis determination and differentiation” Does Figure 3 D (page 42) show the failure of commitment?

Page 12, regarding Fig4B. The authors state “When all XY Z-del gonadal cells are analysed, there is a strong trajectory of PS1 cells”. How is “strong trajectory” measured. Is it a quantitative assessment or subjective? Is it based on how thick the arrows are in Fig4 or how many of them are in the same direction in the PS1 cluster?

Page 43, figure 4 – all six plots have the same name in the figure and do not match the figure legend. The figure would benefit from annotation of different clusters. These RNA velocity trajectory plots could be supplementary material.

Fig5: this figure hard to interpret. Could this be condensed with excess being relegated to supplementary? The red spots, which represent “cells with a similar GCI and SCI score.” How is “similar” measured?

Supplementary Fig S5: Pax8 cells are supposed to be in red, however it is difficult to see which cells are / are not red. When the authors refer to this figure describing Pax8 expression, it’s hard to relate the text to the evidence show in the figure. In contrast, FigS8 and S9 show the red signal (Sox9 and Col18a1 respectively) far more clearly.

The authors present several UMAPs within the paper. It’s not clear which UMAPs contain what data. Sometimes it’s all timepoints and genotypes (Fig1), at other times it’s a B6J CD1 integration (Fig2). Then just the granulosa cluster (Fig3) but is this still the B6J + CD1 integration? Velocity is calculated in Fig4 for select UMAP clusters and genotypes, but from which UMAP? B6J only or B6J + CD1 integration?

The figures S8 and S9 are relevant for the main figure as they show control staining and a gene pair identified as a signature in this dataset.

Scale bars are missing in S5, S8 and S9 figures.

Minor comments

The authors refer to “sensitised” mice in the introduction. They should give a more comprehensive explanation on what that means when first mentioned in the text. It is not clear until later in the paper what the authors mean by this when comparing B6 to CD1.

Relevance of doing B6 to CD1 comparison for baseline establishment as a major part of the manuscript?

The purpose of using machine learning to score cell identity? Furthermore, only using an arbitrary number of 50 marker genes, rather than more commonly used module-scores? The significance, relevance and difference of using a chosen method over mentioned conventional approaches should be explained further.

The authors may want to consider using color-blind friendly colors for their figures S5, S8, S9.

The manuscript would benefit from better sectioning of results with sub-headings.

© 2024. Published by The Company of Biologists under the terms of the Creative Commons Attribution License (https://creativecommons.org/licenses/by/4.0/).

In discussion section on page 16, paragraph starting “Using the same approach, of calculating a score” Znrf3 KO is mentioned, whereas in other text of the manuscript it implies the mutant is Znrf3-deficient. Can this difference between deficiency and knockout be clarified and adhered throughout the text?

“As predicted, when compared to XX WT (Fig. S5d), higher levels of Pax8 expression were observed in the XX mutant tissue along the length of the gonad, with highest levels still observed closer to the mesonephros (Fig. S5e, f)” – the XX mutant should be defined.

Mentioned cell cycle relevance to this study should be explained.

In discussion, page 16, sentence starting: “Moreover, by using transcriptomic signatures of Sertoli cell and granulosa cell identity” – this sentence requires clarification whether both pro-Sertoli and pro-granulosa genes are expressed at a higher level in B6J strain according to the data shown in this paper.

First revision

Author response to reviewers' comments

Reviewer 1 Comments for the Author:
I have a few minor comments:

1. When describing in the introduction the pro-female factors it may be nice to also include RUNX1 (Yao's lab) and the more recently characterised WT1(Chaboissier lab).

We thank the reviewer for this suggestion and have added these genes to the introduction together with the relevant references. The genes have also been added to the marker dot plot in figure 1B.

Page 3, line 56: In the absence of *Sry*, canonical WNT signals direct supporting cell precursors towards a granulosa cell fate (Pannetier, Chassot et al. 2016). **Moreover, in the presence of an overexpressed WT1, transcription factors FOXL2 and RUNX1, were also found to assist supporting cell precursors towards a granulosa cell fate (Yao et al. 2019, Chaboissier et al. 2023).**

2. In figures S8 and S9 - please improve the labelling of the figures: please add scale bars and mention in the figure the stage of the gonads. The D in both (bigger magnification)- is missing the labelling of the RNA-scope markers.

We thank the reviewer for suggesting improvements to the RNA-score images. We added the scale bars and markers to the images. All the images are from the same stage, 12.5dpc, this is noted in the figure legends. We’ve added stage and strain to the image headings for clarity.

Figure S9. (left) VisualisationAnalysis of *Sox9* and *Fst* in B6J wild-type control (+/+) and XY *Z*-del (-/-) gonads at 12.5 dpc.
Figure S10. (right) Visualisation Analysis of *Col18a1* and *Sulf1* in B6J XY WTwild-type control (+/+) and XY *Z*-del (-/-) gonads at 12.5 dpc.

© 2024. Published by The Company of Biologists under the terms of the Creative Commons Attribution License (https://creativecommons.org/licenses/by/4.0/).

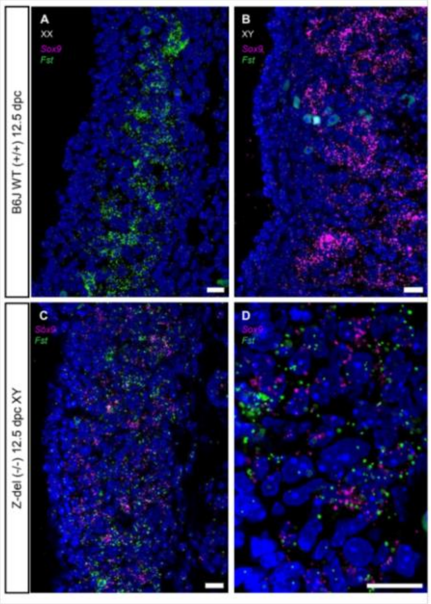

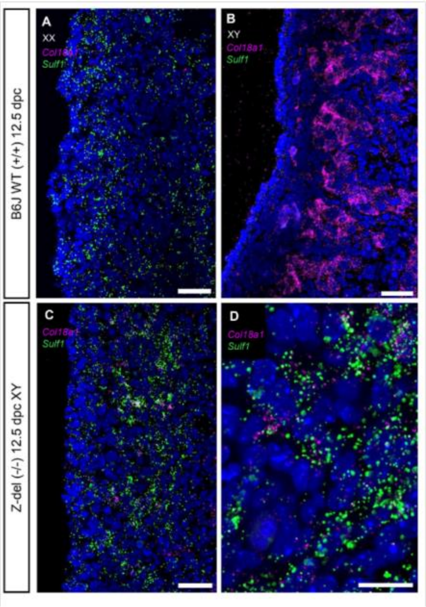

3. In figure S5- again- scale bars and stages but also in G- the labelling is not clear- It is not clear that these are ZNRF3 KO and it is a Sox9 in situ. The figure should be self-explanatory even without the figure legend.

We thank the reviewer for suggesting improvements to figure S5 (now S6). Scale bars were added to S5 A-F and the title “WT and Z-del 13.5dpc *Sox9* in situ” was added to S5G for clarity. We’ve also added stage and strain to the RNAscope image headings for clarity.

Figure S6. *Pax8* expression is elevated in mutant ovarian tissue but no overt rete abnormalities are observed.

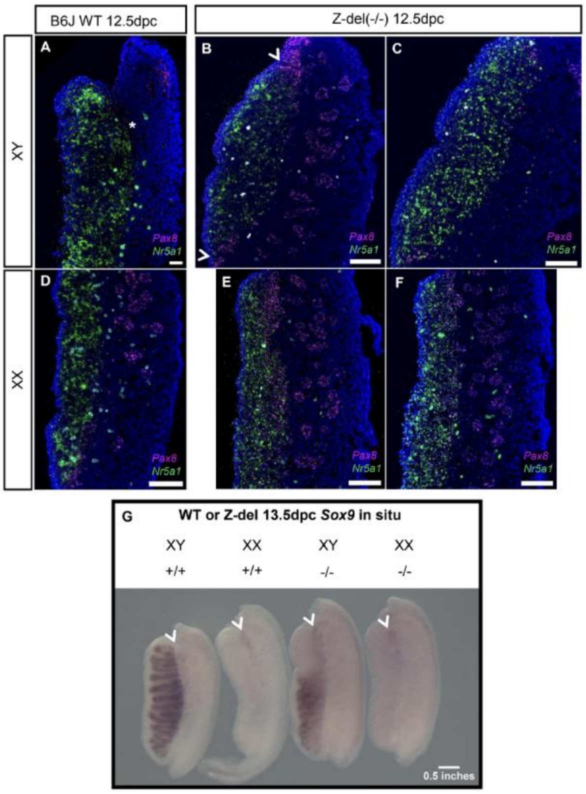

Reviewer 2 Comments for the Author:

© 2024. Published by The Company of Biologists under the terms of the Creative Commons Attribution License (https://creativecommons.org/licenses/by/4.0/).

I have only minor suggestions and amendments.

1. It was interesting to see the differences in Wnt and Lef expression in the different genetic backgrounds. I found myself wondering about the single cell expression of Znrf3 and other pathway members in both the WT/mutant groups of each genetic background. Some of these are shown for example in Figure 1, but could expression across cell types (UMAP or dot plot) be provided for Znrf3, Rnf43, Fzd and Wnts as well as downstream effectors in both genetic backgrounds (WT and mutant). Are there differences in expression of these that are cell lineage-specific?

Unfortunately, we cannot draw conclusions from the expression of the Z-del, since the mutation is a functional KO generated by deleting the RING domain and still produces protein and RNA (Koo et al. 2012, Hao et al. 2012). We feel that showing the expression in the Z-del may be misleading, leading some readers to think that the ZNRF3 protein may still be functional. Hao et al. 2012 have shown that it is a functional KO that produces similar results to a Znrf3 siRNA knockdown in cell lines. We've included a violin plot showing Znrf3 and Rnf43 expression in the B6J WT and Z-del in the different supporting cell clusters. In Harris et al. 2018, they found that the main difference in Znrf3 expression in B6 WT was mainly found at 13.5, which agrees with what we found, with the main differences between XX WT and XY WT found at 14.5. This information is here for your perusal but will not be included in the manuscript.

Figure A. Expression of Znrf3, Rnf43, and Fzd9 in supporting cells of B6 XY WT, XX WT, and XY- del at all time points.

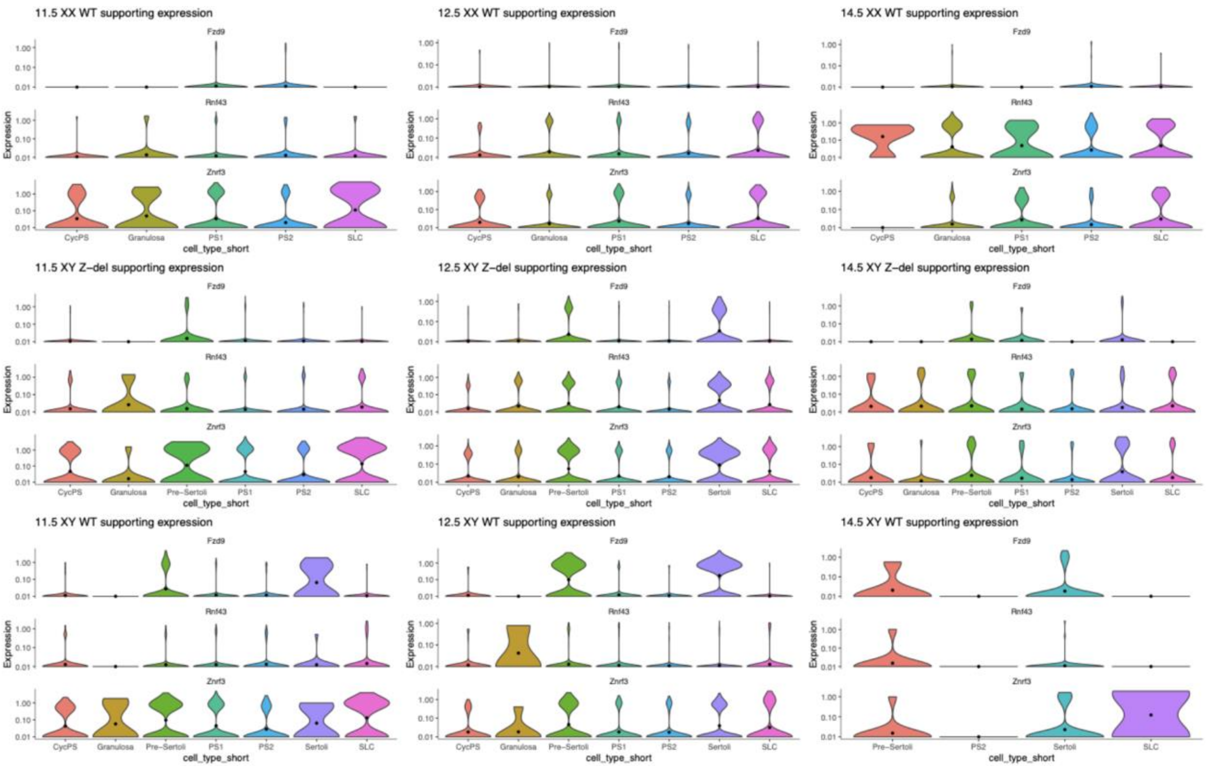

We agree with the reviewer, the differences in WNT pathway genes are intriguing and thank the reviewer for the suggestion. However, there are many WNT associated genes and we were mindful not to overwhelm the manuscript because this is not the main focus of the study. We put a brief description in the results, a new figure showing the top 10 differential Wnt genes at 12.5 dpc in individual supporting cell clusters (below), a new table of all differentials between Z-del and XY WT at 12.5dpc, Table S4, added to the Methods and new legends.

Page 10, line 230:

© 2024. Published by The Company of Biologists under the terms of the Creative Commons Attribution License (https://creativecommons.org/licenses/by/4.0/).

Differential expression analysis of Wnt4, known to be dysregulated in the absence of Znrf3 in gonad development (Harris, Siggers et al. 2018), and LEF, a transcription factor WNT activates, are significantly highly expressed in XY Z-del compared to XY WT at all time points in supporting cells (Fig. S4). To learn more about Znrf3's association with the Wnt pathway we undertook differential expression analysis of supporting cells in XY Z-del versus XY WT. Eighty Wnt pathway genes (Kanehisa et al. 2022) were significantly dysregulated at 12.5dpc (table S4). In XY Z-del 47 Wnt genes were upregulated and 33 downregulated compared to XY WT. The top 10 differential Wnt genes were examined further in the supporting cells separately (Fig S5). Only two genes, Fzd9 and Plcb2, showed XY lineage specific differences. These results show there is a plethora of significant canonical and non-canonical WNT pathway genes affected by Znrf3 KO that warrant further analysis to elucidate their function.

Figure S5. Top 10 differentially expressed WNT pathway genes in the supporting cells between, B6J XY WT and XY Z-del at 12.5dpc.

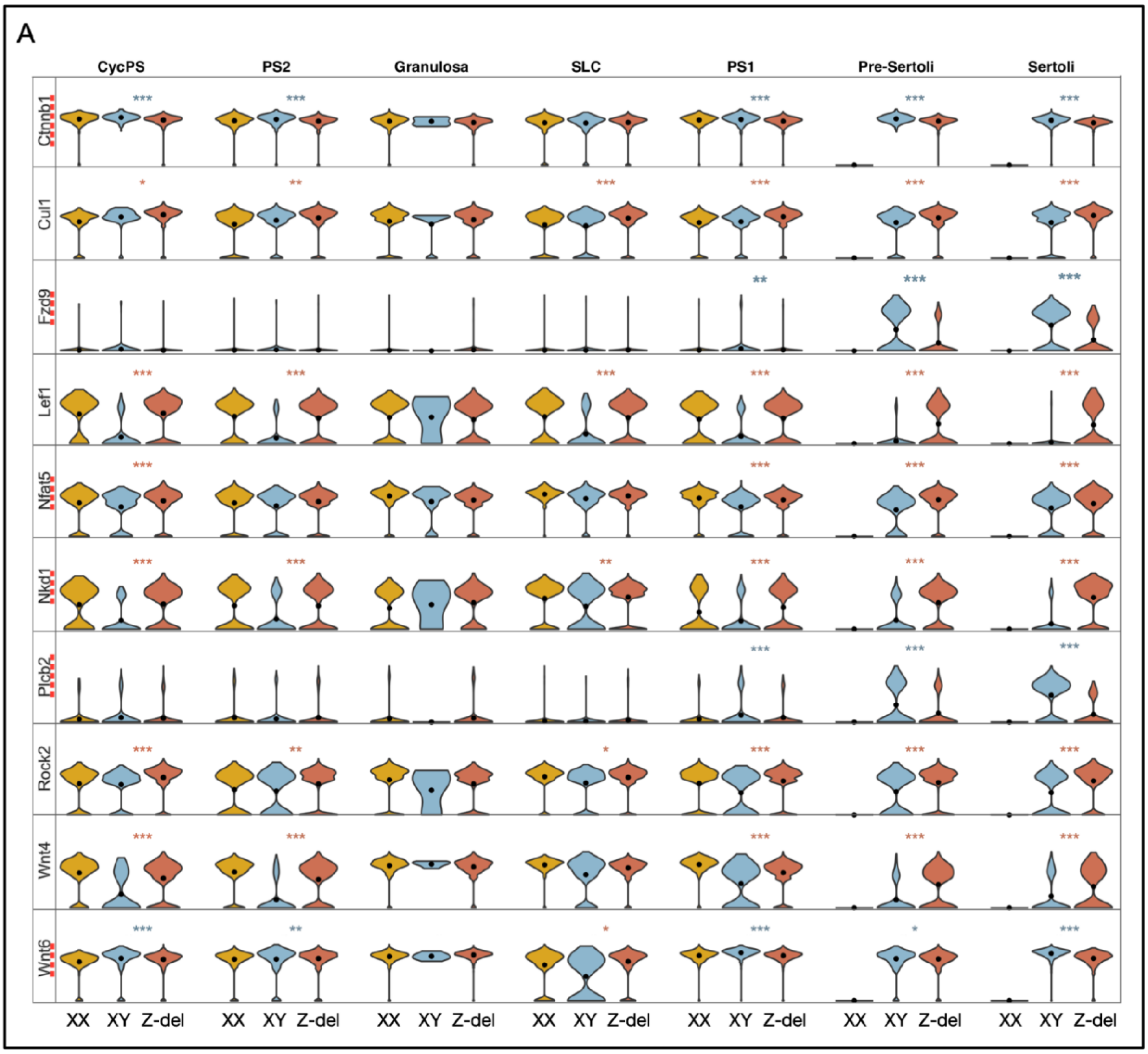

2. The differences observed of Sertoli cells in the G1 cell cycle phase in the Z-del gonads compared to WT made me wonder about the total number of resulting Sertoli + Granulosa (or in fact total number of any somatic cells) in these gonads. I could not see this info provided in any of the tables – but it would be worth commenting on. Is there an overall change in cell numbers? Or just Sertoli proliferation? Are cells committing but not proliferating? Is this exacerbated over time?

We thank the reviewer for their suggestion, and we have added the overall cell numbers per

© 2024. Published by The Company of Biologists under the terms of the Creative Commons Attribution License (https://creativecommons.org/licenses/by/4.0/).

sample, cell- type to table 1. Table 1 contains the cell proportions per sample and cell-type therefore we thought this was an appropriate placement. There is a change in cell numbers per sample however due to the nature of all single-cell experiments we think this reflects technical artefacts, therefore in the manuscript we have mainly presented proportions.

We thank the reviewer again for the next set of questions. They are very involved and in some cases our data can provide an answer in other cases our data is not enough to provide a comprehensive answer.
We have outlined our thoughts below and adjusted the manuscript slightly.

However, the ratio of cell proportion in the XY Z-del Sertoli at 12.5dpc relative to the Pre-Supporting cells is much lower, ~1:1 compared to ~5:1 in XY WT. Contrastingly, XY WT Sertoli make up >60% of the supporting cell clusters, compared to ~5-10% in the XY Z-del and even in more testis-like samples (GX04 in Fig3E) the XY Z-del cluster does not recover the same proportion of Sertoli. This data supports our theory Z-dels cells fail to commit to the Sertoli cell fate. Other proportional data support this theory such as the increase in PS1's (q = 1.25x10-33) and reduction in Pre-Sertoli at 11.5 dpc (Fig3B). It is possible the introduction of XY Z-del Granulosa cells to the mutant pre-supporting cell clusters could account for some reduction in Sertoli cell proportion, however it is unlikely that the relatively small proportion of XY Z- del Granulosa cells fully accounts for the large proportion of Sertoli cell loss.

However, the lack of XY Z-del Sertoli at later stages does contradict how nearly all XY Z-del cells are actively cycling at 12.5 dpc and 14.5 dpc. For example, XY Z-del Sertoli at 12.5dpc have a higher proportion of cells in the G2M and S phases (92%), i.e. cycling phases, than XY WT Sertoli (39%). The phenotype is not exacerbated over time, the proportion of cycling Sertoli is similar at the different stages (92% at 12.5 dpc compared to 85% at 14.5 dpc), while the overall proportion of Sertoli cells increases by only a moderate amount (Fig 3B). These results again suggest a failure to commit to Sertoli throughout all timepoints, however the cells that do achieve a Sertoli cell fate seem to be proliferating. The explanation for these results remain unclear and unfortunately the absolute number together with the cycling phenotype cannot be determined without further single-cell methods such as lineage tracing.

Lastly, later in the manuscript we show the emergence of double positive cells, the backup of cells in the pre-supporting clusters, the transition of Sertoli cells from a high SCI score to a low SCI score and an increase in GCI score, as well as the aberrant cell trajectory at the pre-Sertoli and Sertoli cell cluster.
These results further illustrate a failure of commitment to the Sertoli cell fate.

To clarify the relationship of the cycling phenotype and the failure to commit theory, we have included some extra text to the manuscript, in the Results and the Discussion section, as well as a table in supplementary (Table S3) with numbers and proportions of cycling phases for all stages and supporting cell types in WT and Z-del.

Results, Line 221: Interestingly, XY Z-del Sertoli cells are only rarely (4.1%) found in the G1 cell-cycle phase, (Fig3C) **compared to XY WT Sertoli (52.3%). In contrast, Z-del pre-supporting clusters have similar distributions of cells in the G1 phase to XY WT, both >85% (table S3). However, despite the majority of XY Z-del Sertoli at the later stages actively cycling, the XY Z-del cluster has a disproportionate reduction in Sertoli cells compared to XY WT (e.g. XY WT Sertoli is >60% of the supporting cell clusters, compared to ~5% in the XY Z-del at 12,5dpc). The lack of cells committing to the Sertoli cell fate in Z-dels, despite their cycling nature remains unexplained.**

Discussion Page 18 Line 404: The proportions of different gonadal cell-types are altered in XY Z-del mutant gonads, with **fewer Sertoli,** increased levels of supporting cell precursors (PS1 cluster) at all stages, XY granulosa cells developing and larger numbers of supporting-like cells (SLCs). **These altered proportions are not consistent with the cycling properties of the XY Z-del Sertoli cluster, which is actively proliferating but not significantly increasing in proportion.** These altered proportions are however consistent with an analysis of cell trajectories using RNA velocity, indicating future fates not reflecting robust Sertoli ….

© 2024. Published by The Company of Biologists under the terms of the Creative Commons Attribution License (https://creativecommons.org/licenses/by/4.0/).

3. I understand the focus here is on somatic gonadal cells, however I did find it interesting that in Figure 2B – Germ cell 2 cluster missing in B6J XY c.f CD1 XY. It isn't totally clear to me what the different Germ cell clusters (1,2,3) are here, but this may also be evidence that in addition to B6J showing "feminization" that there may also be delayed maturation (does Germ cell 2 represent those undergoing mitotic arrest?). Similarly, Germ cell pop 3 is missing at E12.5 in XX B6J. Why?

We agree with the reviewer, the disappearance of the Germ 2 cell cluster in B6J is interesting and not discussed in our manuscript. Our manuscript focuses on the XY supporting cells although we are happy to provide the sequencing data to the scientific community for further analysis of other cell-types. To this end we did not investigate the XX lineage cell, as mentioned by the reviewer. However, prompted by your suggestion, we have conducted here a more detailed exploration of the Germ cells.

We have amended the annotations of the Germ cells in figure 2A, from Germ 1, 2 to Early Germ and Germ 3 to Late Germ. This is based on our data showing Germ 1 and 2 consists of mostly 10.5, 11.5, and 12.5dpc, while Germ 3 is mostly 13.5, 14.5, and 16.5dpc, in agreement with the annotations from Mayere et al. 2022. Late Germ is also defined by higher Stra8 expression, which isn't highly expressed in XX B6J until 14.5 dpc (part of fig 2A shown below).

We looked at the expression over time in the germ cell clusters, in B6J and CD1 XX and XY for nine genes associated with germ cell development, Cfc1, Cxcr4, Dazl, Dnmt3l, Nanos2, Nodal, Smad4, Stra8, and Tfap2c (Ref) (Figure A). We focused on 11.5 and 12.5 as they were the comparable timepoints and carried out a differential analysis, classifying differential genes as those with a q-value > 0.05.

For the mitotic genes Cfc1, Cxcr4, and Tfap2c, at 11.5 dpc Cxcr4 is up in the XX CD1 early germ vs. XX B6J, but down in XY CD1 vs. XY B6J. At 12.5 Cfc1 is up in XX CD1 early germ vs. XX B6J, and Tfap2c is down in XX CD1 vs. XX B6J, but up in XY CD1 vs. XY B6J. While the mitotic genes show some differences between B6J and CD1 they are not consistently different across strains. Furthermore, XY germ cell genes Nanos2 and Dmntl3 show no differences in the time points examined, and are generally expressed at later timepoints which can't be compared.

Nodal however, a regulator of germ cell differentiation, is consistently down in CD1 vs. B6J at 11.5 and 12.5. Moreover, the initial expression patterns of Stra8 in the XX (12.5 in CD1 vs. 14.5 in B6J) and Dazl in the XY (11.5 in CD1 vs. 12.5 in B6J) indicate a delay in the B6J strain. This correlates with the presence of the late germ cluster at 12.5 dpc in CD1 but not in B6J. While these results are very interesting, we feel that this is outside the scope of the paper, and further in-vivo and in-silico analysis is required to confirm this strain specific expression

Figure 2. Transcriptomic comparison of sex determination in the CD1 and B6J mouse strains. A) Combined CD1/B6J UMAP showing constituent cell-types (left), strain of origin (middle) and stage of origin (right). The dotted lines group multiple sub-cell-types into one larger cell-type.

© 2024. Published by The Company of Biologists under the terms of the Creative Commons Attribution License (https://creativecommons.org/licenses/by/4.0/).

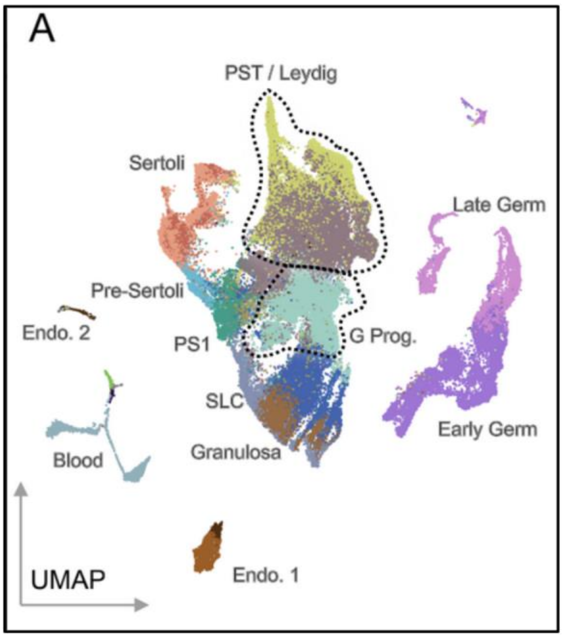

Figure B: Expression of Germ cell associated genes in early and late Germ cell clusters, in B6J and CD1

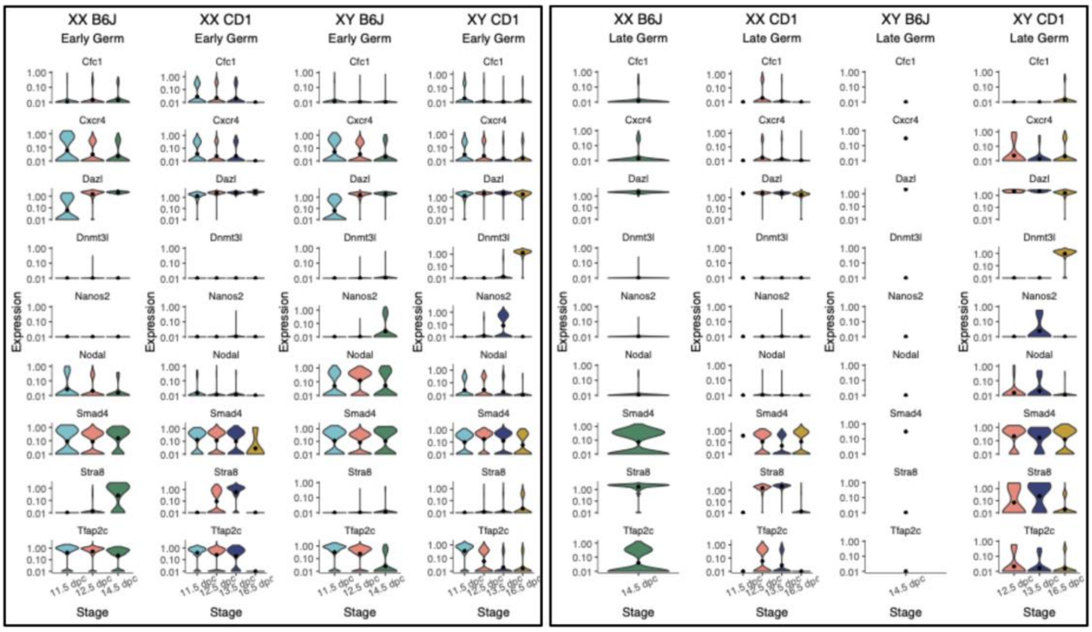

Minor queries and amendments:

1. Although it may be explicit in the text, each Figure and Figure legend should note the genetic background represented. E.g. Figure 1 – BL6.

B6J nomenclature has been added to the figure legends and tables where relevant for consistency. Below shows the amended titles of the Figures:

Figure 1. Single-cell RNA sequencing (scRNAseq) analyses of gonadal sex determination in C57BL/6J (B6J) wild-type and Z-del mouse embryos.

Figure 2. Transcriptomic comparison of sex determination in the CD1 and B6J mouse strains.

Figure 3. The impact of loss of Znrf3 function (Z-del) on gonadal cell transcriptomes in B6J

© 2024. Published by The Company of Biologists under the terms of the Creative Commons Attribution License (https://creativecommons.org/licenses/by/4.0/).

Figure 4. Predicted alteration to cellular differentiation trajectories in B6J XY Z-del gonads.

Figure 5. Pervasive disruption to attainment of cellular sex identity in B6J XY Z-del gonads.

Figure S3. Combined B6J and CD1 datasets (at 12.5 dpc) and supporting cell sex identity in these two strains.

Figure S4. Wnt gene expression in supporting cells of B6J XY wild-type (WT), XX WT and XY Z-del gonads at three stages (11.5, 12.5 and 14.5 dpc).

Figure S5. Top 10 differentially expressed WNT pathway genes between XY B6J WT and XY B6J Z- del and their expression in the supporting cells at 12.5dpc.

Figure S9. Analysis of Sox9 and Fst in B6J wild-type (+/+) and XY Z-del (-/-) gonads at 12.5 dpc.

Figure S10. Analysis of Col18a1 and Sulf1 in B6J wild-type (+/+) and XY Z-del (-/-) gonads at 12.5 dpc.

Figure S11. UMAPs showing location of B6J double-positive (DP) cells.

Fig. S12. UMAP of methanol-fixed and non-fixed B6J gonadal cells.

2. Page 6 results/methods – is n gonads or gonad pairs?

It is gonads, we have changed the text to reflect this, below:
Results Page 6 Line 123: “We examined the cellular composition of **individual** wild-type (WT) XY **gonads** (n= 10), ….”

3. Figure 2A. The different cellular lineages are clear and represented in Figure 1 however in Figure 2A it isn’t clear what Germ 1, 2, and 3 refer to and what the gene expression profiles defining these are.

We thank the reviewer for this suggestion, we believe this is fully answered in the third major revision above.

4. Need to be consistent with case used for Figures and Figure legends (A vs.a)

Uppercase letters have been added to all the Figures and Figure legends.

5. Figure 2 C. Consider expression of other WNT pathway markers too including Znrf3?

We thank the reviewer for this suggestion, we believe this is fully answered in the first major revision above.

6. Figure 2 D. I found this figure difficult to interpret. Consider labelling the four subplots/quadrants ?

We thank the reviewer for spotting this oversight, stage and genotype labels are now added to the figure.
Figure 2D. Plots of GCI (y-axis) and SCI (x-axis) scores found in cells from the B6J and CD1 supporting cell lineage datasets.

© 2024. Published by The Company of Biologists under the terms of the Creative Commons Attribution License (https://creativecommons.org/licenses/by/4.0/).

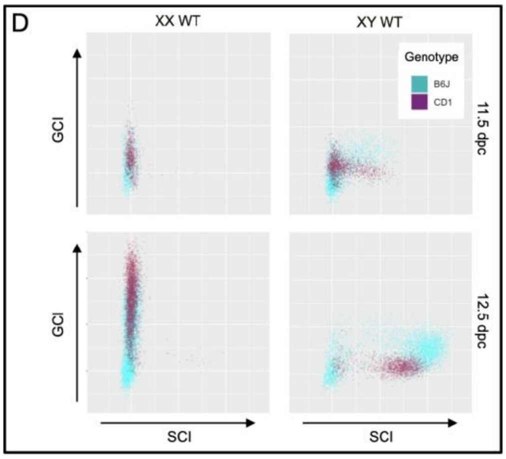

7. Figure 4 A. The figure legend says XY WT but in Figure it says XY Z-Del 12.5 dpc S12. In fact, this heading is used for all velocity plots in this Figure – assuming this is an error, need to revise to indicate the presence of different genotypes and or different samples.

We thank the reviewer for spotting this oversight, the labels are now corrected and added to the figure.

Figure 4A. RNA velocity trajectories shown on UMAP of B6J XY WT, which combines samples from all stages.

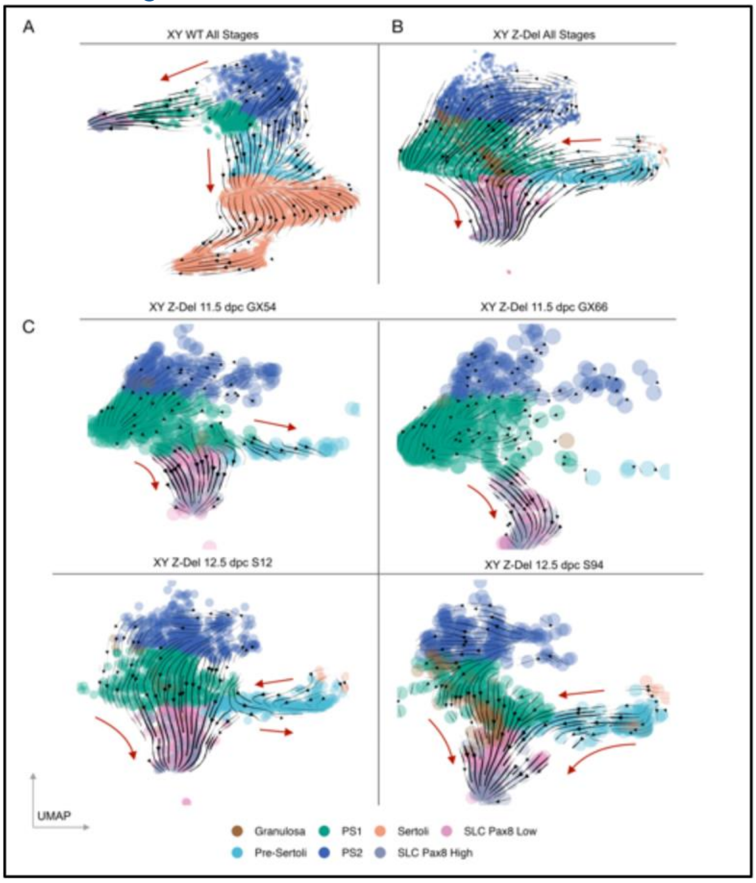

8. Figure S6C. This plot appears to be exactly the same as S6B? Just Amh again? Revise to show Foxl2?

We thank the reviewer for spotting this, the figure is updated with the Foxl2 plot.

© 2024. Published by The Company of Biologists under the terms of the Creative Commons Attribution License (https://creativecommons.org/licenses/by/4.0/).

Figure S7. Expression of Sertoli (*Amh*) and granulosa marker (*Foxl2*) in supporting cells plotted against GCI and SCI scores. A) *Amh* expression is prominent at 12.5 and 14.5 dpc in B6J XY wild-type (WT) cells with high SCI scores (Sertoli cells). Many fewer cells achieve *Amh*-positive Sertoli cell status in XY *Z*-del gonads. B) *Foxl2* expression marks cells with high GCI and low SCI scores in XX WT gonads at all stages. Large numbers of *Foxl2*-positive cells are still observed at 14.5 dpc in XY *Z*-del gonads, in contrast to XY WT.

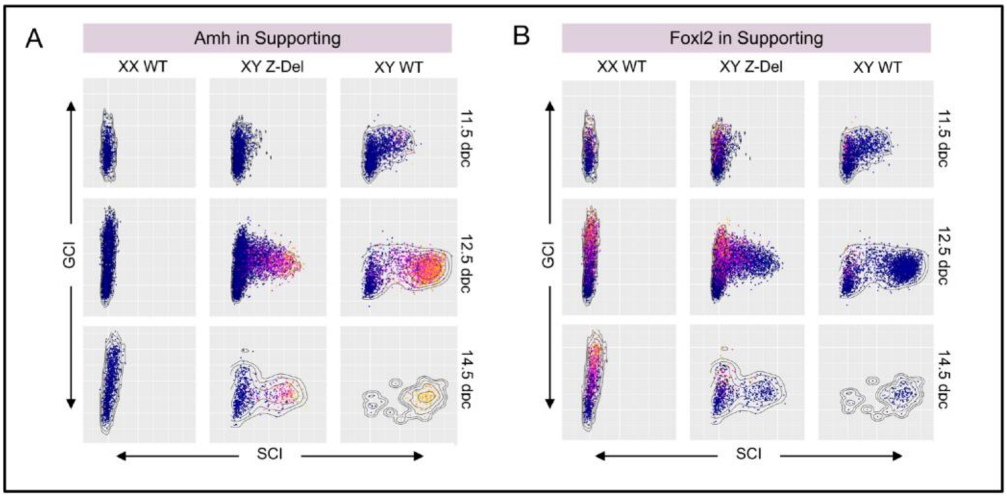

Reviewer 3 Comments for the Author:
Major comments:

1. Currently, the study's scope is narrow and highly descriptive, the analysis lacks clear relevance and context. There are issues with grammar/sentence structure and following the narrative is challenging, and the nomenclature is inconsistent (e.g. supporting cell precursor vs pre-Sertoli and pre-granulosa). There is potential to expand the discussion on other candidate genes affected by Znrf3 KO.

We are disappointed the reviewers did not seem to appreciate the narrative and style of the manuscript. However, we feel heartened that the other two reviewers thought differently and to quote one reviewer, 'very nicely written, clean and concise and very fluent to read and the manuscript will be of a broad interest to reproductive biologists and those who use murine models of sex development'. We have taken great care to write a thought-provoking and clear manuscript, and feel a complete rewrite would be unwarranted here. For example, it is commonplace to use supporting cell precursors, pre-Sertoli and pre- granulosa interchangeably in the developmental sphere and are therefore reluctant to change this.
However, we think all the reviewers comments are constructive and helpful, we have tried to complete the comments fully, as such, we are confident our amendments have improved the manuscript by adding further clarity, brevity and cohesion.

We thank the reviewer for their suggestion to expand the discussion to other candidate genes affected by the Znrf3 KO. This was also noted by another reviewer, therefore we have expanded our narrative to include more discussion on the Wnt genes which are associated with Znrf3 and already part of the manuscript. Differential analysis of genes expressed between XY WT and Z-del at 12.5dpc are shown in table S4 and the top 10 significantly differentiated expressed Wnt genes shown in figure S5.
Please refer to Reviewer 2, point 1 revision and response for further explanation.

2. There are concerns related to reproducibility, including the GitHub link provided by the authors, which is currently empty.

The code is now added to GitHub, https://github.com/MichyCode/Gonadal-SIS-Method.

And missing supplementary bioinformatic methods.

© 2024. Published by The Company of Biologists under the terms of the Creative Commons Attribution License (https://creativecommons.org/licenses/by/4.0/).

All methods are and were in the Methods section of the manuscript. Any reference to Bioinformatic Methods in the manuscript has been removed.

Furthermore, the authors describe using a dataset containing 68,000 cells. This dataset contained three genotypes (XX, XY, XY Znrf3 KO), at three timepoints (11.5, 12.5 and 14.5), however there's no breakdown of how many cells there were per genotype / timepoint.

We thank the reviewer for their suggestion, Table 1 consists of cell proportions per sample, genotype, stage, we have now included the overall cell numbers to this table. Also, we should have put near 68,000 cells because the precise number is 67,605. This has now been updated in the introduction and a legend.
Line 126: "around 68,000 cells,"

Line 542 (Figure 1): "approximately 68,000..."

Are all three genotypes and timepoints represented equally in terms of cell number? They give a breakdown of proportions per cluster in Fig 2B, however it's harder to interpret if we don't know what the timepoint proportions are.

All three genotypes are not represented equally, for example there are more gonads, therefore more cells for 12.5dpc samples compared to 11.5dpc samples. Consequently, we presented proportions instead of absolute cell numbers. 12.5dpc is the stage where the gonads differentiate, following the expression of SRY (Hacker et al. 1995), and as such we wanted to capture as much information as we could about this critical time point, especially in the XY Z-del. The total number of cells per sample, stage and cluster are now in table 1, refer to comment above.

Clarification is required on the filtering of LQ cells. They state that Seurat was used to remove low quality cells based on genes expressed, counts etc. Under "Filtering of Low-Quality Cells" they describe that thresholds were changed per sample due to sequencing depth, but then give hard thresholds, i.e. "Cells with UMI counts of <2% and >98% were removed", so were these thresholds applied to all cells, regardless of their sequencing depth?

We thank the reviewers for noticing the intricacies of our methods. To explain, we initially used the same QC threshold on every sample individually (this should account for differing sequencing depth), however, we found the cells in the lower 5th percentile of counts clustered to produce small isolated clusters, due to low counts only. Therefore, we used a second global threshold over the whole dataset to further exclude low quality cells. We realise this was a stringent strategy but wanted to be sure all low quality cells were removed from the analysis. To clarify any confusion we have adjusted the Bioinformatic methods, below.

Methods Page 28 Line 647: **Cells falling outside quality control thresholds were removed from the dataset first on a per sample basis and then for the whole dataset. Per sample cells with; UMI counts of <2% and >98% of the total were removed, genes expressed in <2% and >98% of the total were removed and mitochondrial RNA% expressed in <2% and >95% of the total were removed.**
After further inspection we noticed low quality cells were still present, therefore a global filter was applied across the whole dataset to exclude cells in the bottom 5th percentile of counts, i.e., cells with less than ~5000 counts.

Following on, in the section "Cell clustering and cell-type annotation", they then apply more filtering by removing "Cells within the bottom 5% percentile of counts (~5000 counts)", with the aim of removing small UMAP clusters. But doesn't this mean two rounds of LQ filtering have occurred? Usually, the initial LQ filtering should be enough as they already excluded low counts cells, but then they do it again to make the UMAP?

Please refer to the answer in the above comment.

3. A clarification is required on the background that B6J strain was compared to in this study. Mayere et al study used mixed 129/CD1 strain rather than pure CD1 background. Furthermore, Mayere et al, 2021 bioRxiv reference is missing from bibliography.

© 2024. Published by The Company of Biologists under the terms of the Creative Commons Attribution License (https://creativecommons.org/licenses/by/4.0/).

We thank the reviewer for noticing this oversight. The correct nomenclature has been added to the first appearance of CD1 in the main text and in the methods, below. Mayere *et al*, is now published in Science advances and the reference was in the bibliography shown below.

Results Page 7 Line 159: gonadal dataset generated in **129**/CD1**(from here referred to as CD1)** strain
Methods Page 30 Line 684: Count data from Mayere *et al*. 2022, containing **129**/CD1
Reference: Mayère, C., Regard, V., Perea-Gomez, A., Bunce, C., Neirijnck, Y., Djari, C., Bellido-Carreras, N., Sararols, P., Reeves, R., Greenaway, S., Simon, M., Siggers, P., Condrea, D., Kühne, F., Gantar, I., Tang, F., Stévant, I., Batti, L., Ghyselinck, N. B., Wilhelm, D., … Nef, S. (2022). Origin, specification and differentiation of a rare supporting-like lineage in the developing mouse gonad. Science advances, 8(21), eabm0972. https://doi.org/10.1126/sciadv.abm0972

4. The figures require major attention, for example:

Fig1C: The large dotplot showing marker expression per UMAP cluster does re-enforce that the clusters have indeed been annotated correctly, however this kind of figure would be better suited in supplementary.

There are several reasons why the dot-plot is in figure 1; one, the purpose of figure 1 is to describe our dataset and an introduction into the annotation of the clusters. Two, there are limited publications on gonad development and we believe many researchers will find a front and centre dot-plot useful. Therefore, we respectfully thank the reviewer for their suggestion, however we strongly believe this figure should remain in its current position.

Page 40, figure 1 C – it would be easier to interpret this dot plot if it would highlight appropriate sections for specific populations or marker genes.

We thank the reviewer for this suggestion, we have amended figure 1C as suggested and believe it is easier to interpret now.

Figure 1. scRNAseq analyses of gonadal sex determination in C57BL/6J (B6J) wild-type and *Z*-del mouse embryos. C Dotplots detailing expression specific to each cell type.

© 2024. Published by The Company of Biologists under the terms of the Creative Commons Attribution License (https://creativecommons.org/licenses/by/4.0/).

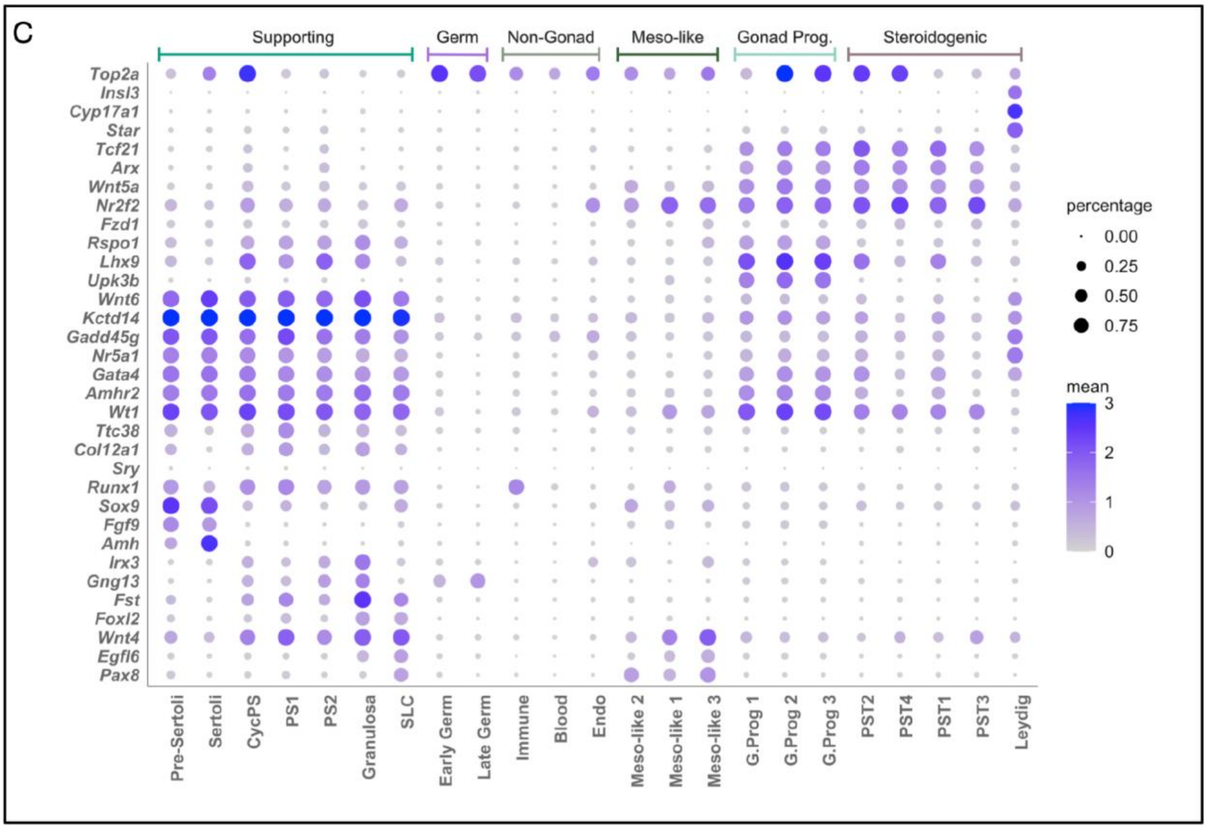

Page 40, figure 1 B – this would benefit from separate UMAPs of different time points. It is unclear if some cells from different timepoints overlap.

On reflection we agree with the reviewer and thank the reviewer for their comment. We did attempt separate UMAPs as suggested however we found it was still unclear to determine what cells co-localise with other cells from disparate timepoints. We then tried many different representations and the clearest representation was decreasing the cell size. We believe you can now see the overlapping cells clearly. In addition, a figure containing 3 separate UMAPs as suggested would be a duplicate of the 3 UMAPs showing supporting cells in figure 3.

Figure 1B. UMAP of all B6J cells coloured by stage (blue = 11.5 dpc, coral = 12.5 dpc, green = 14.5 dpc.

© 2024. Published by The Company of Biologists under the terms of the Creative Commons Attribution License (https://creativecommons.org/licenses/by/4.0/).

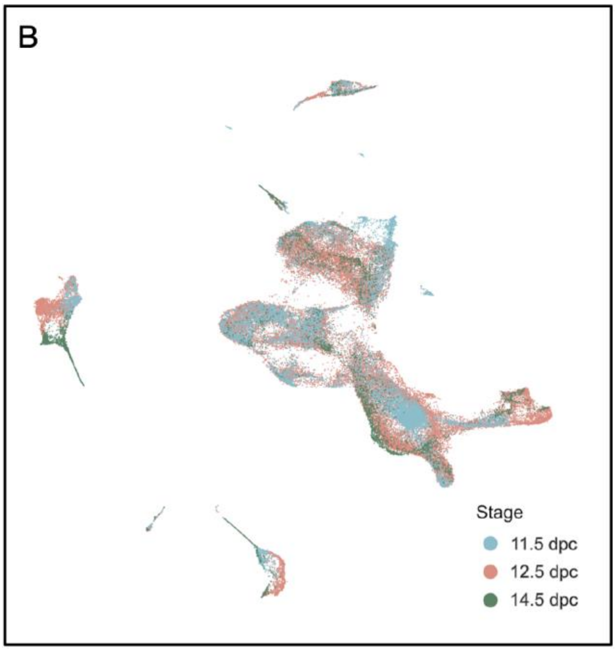

Fig2B: The barplots showing proportions per cluster. There are 18 clusters, which makes interpretation of this figure difficult. Again, would be better suited in supplementary, or simplified by combining numbered clusters, i.e. “Germ1”, “Germ2”, “Germ3” = “Germ”

We thank the reviewer for their comment and agree the barplots may be too granular for interpretation. For visualisation purposes we have simplified the barplots whilst preserving important biological details, by combining some clusters and slightly re-annotating others. Germ1, Germ2 and Germ3 are now Early and Late Germ. PST1, PST2, PST3 and PST4 are combined to create PST. G.Prog1, G.Prog2 and G.Prog3 are combined to create G.Prog. We felt PS1 and PS2 clusters are pivotal to the manuscript and have therefore remained as separate clusters. The adjusted figure is below.

Figure 2B. Proportions of 18 different cell-types in B6J and CD1 datasets at 11.5 (upper) and 12.5 dpc (lower)

© 2024. Published by The Company of Biologists under the terms of the Creative Commons Attribution License (https://creativecommons.org/licenses/by/4.0/).

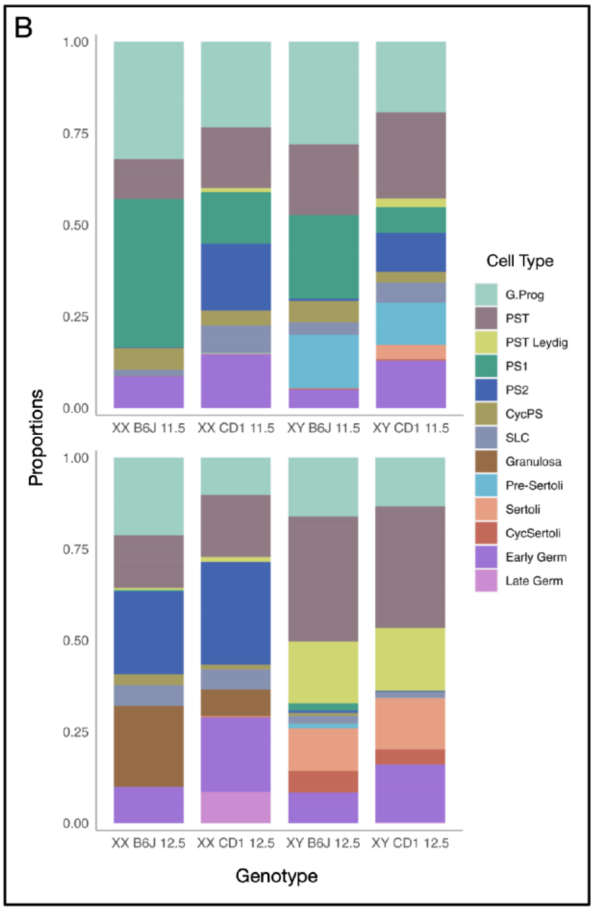

Fig2D: It's not clear what each individual scatter plot represents. For example, the bottom right scatter plot is more SCI than GCI, and the bottom left is low in both SCI and GCI, but what is the relationship between each plot? How are these discrete plot boundaries decided? Page 41, figure 2 D – it is not clear how the scatter plot is divided into quarters and what the significance is.

We thank the reviewer for spotting this slight oversight. Even though an explanation is given in the legends we agree with the reviewer that the image is not clear. We have now added label names to the quadrants. Please refer to Reviewer 2, point 6 for the new figure.

Page 10, "A principal component analysis of these proportions (Fig.3d, Table S3) shows that XY Z-del mutants at 12.5 dpc group with 11.5 dpc XX and XYwild-type samples, suggesting a failure of commitment to testis determination and differentiation" Does Figure 3 D (page 42) show the failure of commitment?

We believe figure 3D does show a failure of commitment to testis determination and differentiation. The PCA (Fig 3D) was constructed from all gonadal clusters and represents the variation in Z-dels compared to the other genotypes. Inspecting this PCA together with the proportions (Fig 3B) we see an increase in Pre-Supporting 1, Pre-Supporting 2 and Granulosa, a decrease in Sertoli and in the PCA, a grouping of 12.5dpc XY Z-dels with 11.5dpc XX WT. Overall this suggests Z-del feminisation and a failure to commit to the Sertoli cell fate.

Page 12, regarding Fig4B. The authors state "When all XY Z-del gonadal cells are analysed, there is a strong trajectory of PS1 cells". How is "strong trajectory" measured. Is it a quantitative assessment or subjective? Is it based on how thick the arrows are in Fig4 or how many of them are in the same direction in the PS1 cluster?

We appreciate the question thank you. We wanted to highlight the trajectory away from Sertoli and towards SLC, however in our eagerness we may mislead the reader by stating 'strong' trajectory. Black arrows indicate direction and thickness indicates speed along the gonadal cell

© 2024. Published by The Company of Biologists under the terms of the Creative Commons Attribution License (https://creativecommons.org/licenses/by/4.0/).

development trajectory. Due to the number of arrows pointing in the same direction a more fitting phase would be a consensus trajectory. We have adjusted the manuscript below:

Results Page 13 Line 290: When all XY Z-del gonadal cells are analysed (Fig. 4b), there is a **consensus** trajectory of PS1 cells, which exist in larger numbers compared to XY WT (Fig. 3b), towards SLCs (Fig 4b, c).

Page 43, figure 4 – all six plots have the same name in the figure and do not match the figure legend. The figure would benefit from annotation of different clusters. These RNA velocity trajectory plots could be supplementary material.

We thank the reviewer for spotting this oversight. The labels have been corrected. Due to the large discussion of this result in the manuscript we would like to leave the figure among the main figures. The labelling has been amended and shown in the above revision. Please refer to Reviewer 2, point 7 for the correct figure.

Fig5: this figure hard to interpret. Could this be condensed with excess being relegated to supplementary? The red spots, which represent "cells with a similar GCI and SCI score." How is "similar" measured?

Figure 5 represents the distribution of gonadal cells in their acquisition to supporting cell fate, described by Sertoli Cell Identity (SCI) and Granulosa Cell Identity (GCI). We subsequently used these scores to compare across genotypes and stage. The main point being the disruption of gonadal development affects multiple supporting cell types in different ways, including the acquisition of low-Pax8 cells leading to higher GCI in the SLCs, a new and prominent cell-type in the gondanal field. The variable SCI score and high GCI score in Sertoli, illustrates on a cellular level the disruption & feminisation of Z-del Sertoli cell development respectively. The emergence of double positive cells refers to the presence of XX and XY pathways in the same cell, to our knowledge this has not been shown previously in a mouse model. To this extent we believe this figure as a whole should be in the main body of the manuscript.

We thank the reviewer for noticing we have not included the methods for double positives, this is now in the manuscript and is below. For similarity, cells are classified as passing the threshold and double positive if the sum of SCI and GCI squared, over the b squared (where b is the 97.5th percentile of GCI in 14.5 dpc XX WT Granulosa), is greater than the difference between SCI and GCI squared, over the scaling factor. Essentially cells with a larger sum of SCI and GCI than the difference between their scores (divided by the scaling factor) will be classified as similar.

Added to methods:

Identification of Double positive cells

Double positive cells have similar SCI and GCI scores. To identify cells with similar SCI and GCI scores, we defined a hyperbola using the equation below where a is a scaling factor of 10 and b is the threshold or boundary of the hyperbola, represented by the 97.5% percentile of GCI scores in 14.5 dpc XX Granulosa WT cells. This returns TRUE if the cell is within the defined region. These cells are called double positive cells.

$$if\ \frac{(\mathrm{GCI}+\mathrm{SCI})^2}{b^2}-\frac{(\mathrm{GCI}-\mathrm{SCI})^2}{a^2}>1\ \wedge abs(GCI*SCI)>0$$

To compare the proportions of double positive cells to non-double positive cells, we used a chi-squared test (with a False Discovery Rate).

Supplementary Fig S5: Pax8 cells are supposed to be in red, however it is difficult to see which cells are / are not red. When the authors refer to this figure describing Pax8 expression, it's hard to relate the text to the evidence show in the figure. In contrast, FigS8 and S9 show the red signal (Sox9 and Col18a1 respectively) far more clearly.

© 2024. Published by The Company of Biologists under the terms of the Creative Commons Attribution License (https://creativecommons.org/licenses/by/4.0/).

We thank the reviewer for the comment, on reflection we fully agree. We have updated the figure to the contrasting colours, green and magenta. We hope this makes the image more comprehensible. Please refer to Reviewer 1, point 3 for the figure.

The authors present several UMAPs within the paper. It's not clear which UMAPs contain what data. Sometimes it's all timepoints and genotypes (Fig1), at other times it's a B6J CD1 integration (Fig2). Then just the granulosa cluster (Fig3) but is this still the B6J + CD1 integration? Velocity is calculated in Fig4 for select UMAP clusters and genotypes, but from which UMAP? B6J only or B6J + CD1 integration?

We agree with the reviewer, there are a lot of necessary UMAPs in the manuscript. Figure 2 is the only figure in the manuscript's main body that has B6J and CD1 data. To reduce the confusion we have added the mouse background strain to all the titles of the legends in the main manuscript, for example Figure 2 clearly states CD1 and B6J (as before) and now Figure 3 states just B6J.

Please refer to Reviewer 1, minor point 1 for the new figure titles.

The figures S8 and S9 are relevant for the main figure as they show control staining and a gene pair identified as a signature in this dataset.

Scale bars are missing in S5, S8 and S9 figures.

We thank the reviewer for noticing this oversight. We have now added the scale bars to the figures. While the figures are relevant by showing staining and gene pairs identified in our dataset, we feel this was a validation and not integral to our main story.

Reviewer 3 Minor comments

The authors refer to "sensitised" mice in the introduction. They should give a more comprehensive explanation on what that means when first mentioned in the text. It is not clear until later in the paper what the authors mean by this when comparing B6 to CD1.

We thank the reviewer for noticing this oversight. We have moved the description of sensitised nature of B6J from the first sentence of the results to the first mention of sensitised B6J in the introduction. Also added reference,Narita et al. 2023 which states that "the B6J strain of mice have fewer SRY expressing cells, and given the description of the mutually antagonistic sex development pathway, fewer SRY expressing cells in this case equates easier feminisation when the pathway is further disturbed".

Introduction Page 4 Line 76: **"a mouse strain sensitised to disruptions to testis determination due to fewer SRY expressing cells (Bouma, Washburn et al. 2007, Narita et al. 2023),"**

Relevance of doing B6 to CD1 comparison for baseline establishment as a major part of the manuscript?

B6J is a mouse strain sensitised to sex reversal, therefore we thought it pertinent to add the CD1 comparison as context before introducing the sex reversal phenotype of XY Z-del gonads at the single cell level. This is already in the manuscript, below. However, beyond this, we thought it maybe a valuable resource for the scientific community, as the developing gonads of the B6J strain has not been previously detailed at the single-cell level, and as noted by reviewer one, "there other mouse strains being generated on the C57BL/6J background".

Introduction Page 4 Line 92: We also compared the landscape of sex determination at single-cell resolution in the sensitised B6J strain with the non-sensitised strain CD1 (Mayere 2021), which allowed us to calibrate the nature of sex reversal in the *Z*-del mutants by understanding the 'feminised' baseline of testis determination in B6J XY gonads.

The purpose of using machine learning to score cell identity? Furthermore, only using an arbitrary number of 50 marker genes, rather than more commonly used module-scores? The significance, relevance and difference of using a chosen method over mentioned conventional approaches should

© 2024. Published by The Company of Biologists under the terms of the Creative Commons Attribution License (https://creativecommons.org/licenses/by/4.0/).

be explained further.

The purpose of using machine learning to score cell identity is two-fold. First, to identify a list of genes that could represent Sertoli and Granulosa cells identity in a non-biased manner. Second, to develop a model to illustrate the level of Sertoli-ness and Granulosa-ness per cell and cluster in any gonad.

In this pursuit, we fit all genes to a GLM to rank them on their ability to positively and negatively discriminate Sertoli and Granulosa cells from those in other clusters (the rank is based on AUC ROC). After the ranking, we built a gene set by sequentially adding genes to a GLM model and assessed whether they improved the model's ability to discriminate Sertoli from other cell types (via an AUC ROC curve), and stopped adding genes when the model found no further improvement. For Sertoli we found the model reached it's peak ability to determine Sertoli with a set of 50 genes (i.e. the point at which adding more genes did not improve our ability to determine Sertoli). Therefore, we used 50 genes for the model with Sertoli. For Granulosa we found that a model of 25 genes had the maximum ability to discriminate, however to maintain consistency, the final model for both used 50 genes. As the module score function does not have the capability to do this, we sought to use ML as above to identify a list.

After the gene list was determined we created a model, representing Sertoli and Granulosa cells within the WT datasets. This model can then be applied to new samples, to score each cell based on their transcriptomic similarity to the desired types - Sertoli and Granulosa. Hence, the AddModuleScore function was not appropriate for our needs. All is detailed in the Methods, the sections are Gene Assessment and Model Building.

The authors may want to consider using color-blind friendly colors for their figures S5, S8, S9.

We thank the reviewer for this comment. In response, we have changed the red to magenta, as the journal suggests, for figures S6, S9, and S10. Please refer to Reviewer 1, revision 1 and 2.

The manuscript would benefit from better sectioning of results with sub-headings. We thank the reviewer for this and have added four section headings to the Results:
Line 116: **Annotation of the gonadal cell clusters**
Line 155: **Comparative analysis of B6J and CD1 gonads**
Line 212: **Failure to commit to testis Determination**
Line 313: **Pervasive disruption to supporting cells**

In discussion section on page 16, paragraph starting “Using the same approach, of calculating score” Znrf3 KO is mentioned, whereas in other text of the manuscript it implies the mutant is Znrf3-deficient. Can this difference between deficiency and knockout be clarified and adhered. throughout the text?

We thank the reviewer for this comment. Here we use the phrases Znrf3-KO and Znrf3-deficient for related but different purposes. We used “Znrf3-deficient” to describe these mutant gonads, meaning the homozygous mutants do not have functional ZNRF3, and “Znrf3 KO” in turn mentions the actual knock out of ZNRF3. This was used incorrectly in the discussion, and Znrf3 deficient should have been used. We have adjusted the line accordingly.

Discussion Page 17, Line: 401: “Using the same approach, of calculating a score for the attainment of Sertoli (SCI) or granulosa cell (GCI) fate, alongside an analysis of cellular trajectories in control and mutant gonads, we show that fetal gonadal sex reversal in *Znrf3*-deficient (Z-del) mice is characterised by the pervasive disruption to cell fate acquisition.”

“As predicted, when compared to XX WT (Fig. S5d), higher levels of Pax8 expression were observed in the XX mutant tissue along the length of the gonad, with highest levels still observed closer to the mesonephros (Fig. S5e, f)” – the XX mutant should be defined.

We thank the reviewer for this comment and have added text to clarify we are referencing the XX Z-del mutant.

© 2024. Published by The Company of Biologists under the terms of the Creative Commons Attribution License (https://creativecommons.org/licenses/by/4.0/).

Results Page 11 Line 274: "As predicted, when compared to XX WT **(Fig. S6D)**, higher levels of *Pax8* expression were observed in the XX **Z-del** mutant tissue along the length of the gonad, with highest levels still observed closer to the mesonephros"

Mentioned cell cycle relevance to this study should be explained.

We thank the reviewer for this comment. We know ZNRF3 regulates WNT signalling, and a role of WNT signalling is it's impact on the cell cycle. Up-regulation of Wnt is known to positively affect proliferation (Liu et al. 2022), therefore the association of Z-del, Wnt and cycling warrants some discussion in the manuscript. Please refer to Reviewer 2, Minor Comment 2 for more information.

In discussion, page 16, sentence starting: "Moreover, by using transcriptomic signatures Sertoli cell and granulosa cell identity" – this sentence requires clarification whether both pro-Sertoli and pro- granulosa genes are expressed at a higher level in B6J strain according to the data shown in this paper.

We thank the reviewer for this question. We have updated the sentence.

Discussion Page 17 Line 394: "Moreover, by using transcriptomic signatures of Sertoli cell and granulosa cell identity, we show that **alongside the heightened** expression of pro-granulosa genes in B6J, **there are** higher proportions of cells also expressing both pro-Sertoli and pro-granulosa genes…"

Second decision letter

MS ID#: DEVELOP/2024/202707

MS TITLE: Gonadal sex reversal at single-cell resolution in Znrf3-deficient mice

AUTHORS: Raissa GG Kay, Richard Reeves, Pam Siggers, Simon Greenaway, Ann-Marie Mallon, Sara Wells, Bon-Kyoung Koo, Chloe MAYERE, Serge Nef, Andy Greenfield, and Michelle M Simon

I am happy to tell you that your manuscript has been accepted for publication in Development, pending our standard publication integrity checks. Where referee reports on this version are available, they are appended below.

Reviewer 1

Advance Summary and Potential Significance to Field:

The authors have addressed all my concerns and I would recommend it for publication in Development.

Comments for the Author

No other revision is needed by me

Reviewer 2

Advance Summary and Potential Significance to Field

In this paper the authors analysed Znrf3 mutant mouse gonads at single cell resolution. This work provided interesting insight into the molecular perturbations behind the Znrf3 phenotype and the observed phenotypic variability.

© 2024. Published by The Company of Biologists under the terms of the Creative Commons Attribution License (https://creativecommons.org/licenses/by/4.0/).

This work provides a significant and novel understanding of sex development of the gonads and how it can be disrupted, which is of interest to all researchers in the sex differentiation field including those working in human reproductive conditions. It also revealed why a B6J genetic background is sensitised to sex development aberrations, which will be of broad interest to reproductive biologists and those who use murine models of sex development.

Comments for the Author

I am satisfied that the authors have addressed all previous comments and suggestions.

Reviewer 3

Advance Summary and Potential Significance to Field:

Comments for the Author

I appreciate the authors attention to my previous comments, and think the ms is improved as a result.

© 2024. Published by The Company of Biologists under the terms of the Creative Commons Attribution License (https://creativecommons.org/licenses/by/4.0/).
